# Comprehensive pathogen identification and antimicrobial resistance prediction from positive blood cultures using nanopore sequencing technology

**DOI:** 10.1186/s13073-024-01416-2

**Published:** 2024-12-02

**Authors:** Po-Yu Liu, Han-Chieh Wu, Ying-Lan Li, Hung-Wei Cheng, Ci-Hong Liou, Feng-Jui Chen, Yu-Chieh Liao

**Affiliations:** 1 Division of Infectious Diseases, Department of Internal Medicine, Taichung Veterans General Hospital, Taichung, Taiwan; 2 Ph.D. Program in Translational Medicine, National Chung Hsing University, Taichung, Taiwan; 3 Rong Hsing Research Center for Translational Medicine, National Chung Hsing University, Taichung, Taiwan; 4 Department of Post-Baccalaureate Medicine, College of Medicine, National Chung Hsing University, Taichung, Taiwan; 5 National Institute of Infectious Diseases and Vaccinology, National Health Research Institutes, Miaoli County, Zhunan, Taiwan; 6 Institute of Population of Health Sciences, National Health Research Institutes, Miaoli County, Zhunan, Taiwan; 7 Department of Biological Science and Technology, National Yang Ming Chiao Tung University, Hsinchu, Taiwan

**Keywords:** Pathogen identification, Antimicrobial resistance prediction, Positive blood cultures, Real-time, Nanopore sequencing, Adaptive sampling

## Abstract

**Background:**

Blood cultures are essential for diagnosing bloodstream infections, but current phenotypic tests for antimicrobial resistance (AMR) provide limited information. Oxford Nanopore Technologies introduces nanopore sequencing with adaptive sampling, capable of real-time host genome depletion, yet its application directly from blood cultures remains unexplored. This study aimed to identify pathogens and predict AMR using nanopore sequencing.

**Methods:**

In this cross-sectional genomic study, 458 positive blood cultures from bloodstream infection patients in central Taiwan were analyzed. Parallel experiments involved routine microbiologic tests and nanopore sequencing with a 15-h run. A bioinformatic pipeline was proposed to analyze the real-time sequencing reads. Subsequently, a comparative analysis was performed to evaluate the performance of species identification and AMR prediction.

**Results:**

The pipeline identified 76 species, with 88 *Escherichia coli*, 74 *Klebsiella pneumoniae*, 43 *Staphylococcus aureus*, and 9 *Candida* samples. Novel species were also discovered. Notably, precise species identification was achieved not only for monomicrobial infections but also for polymicrobial infections, which was detected in 23 samples and further confirmed by full-length 16S rRNA amplicon sequencing. Using a modified ResFinder database, AMR predictions showed a categorical agreement rate exceeding 90% (3799/4195) for monomicrobial infections, with minimal very major errors observed for *K. pneumoniae* (2/186, 1.1%) and *S. aureus* (1/90, 1.1%).

**Conclusions:**

Nanopore sequencing with adaptive sampling can directly analyze positive blood cultures, facilitating pathogen detection, AMR prediction, and outbreak investigation. Integrating nanopore sequencing into clinical practices signifies a revolutionary advancement in managing bloodstream infections, offering an effective antimicrobial stewardship strategy, and improving patient outcomes.

**Supplementary Information:**

The online version contains supplementary material available at 10.1186/s13073-024-01416-2.

## Background

Bloodstream infection (BSI) are marked by positive blood cultures and systemic signs of infection [1]. They occur in community and hospital settings from various sources, affecting diverse patients with different microorganisms [2]. Surveillance studies reveal increasing BSI rates over time [3], with a shift in antimicrobial resistant profiles, particularly the emergence of multidrug-resistant (MDR) microorganisms [4, 5], posing significant therapeutic challenges in BSI management.

Tracking BSI pathogens and antibiotic susceptibility patterns is crucial for guiding empiric antibiotic treatments and enabling precision medicine. However, the process often takes 2–5 days in diagnostic labs [6]. Recently, rapid antimicrobial susceptibility testing (AST) devices like the Accelerate PhenoTest BC system [7–10] and Q-linea ASTar system [11, 12] have emerged for swift phenotypic AST directly from blood cultures, with a turnaround time of 6–7 h [8, 11]. However, this phenotypic analysis offers limited resolution on epidemiology [13–17]. Genomic sequencing holds potential to revolutionize antimicrobial resistance (AMR) surveillance, providing a high-resolution depiction of the AMR evolution and transmission [13, 18].

Oxford Nanopore Technologies (ONT) offers scalable technology for direct analysis of long DNA or RNA fragments. ONT has gained interest for rapid microbial identification and AMR genotyping [15, 19–21], showing success in clinical and simulated BSI scenarios [21]. To address low coverage of causative pathogens due to human DNA abundance, ONT introduced adaptive sampling in the GridION system [22], which enriches target DNA or deplete undesired DNA during sequencing [23]. Although adaptive sampling has been evaluated for enrichment in metagenomic samples [22, 24–26], there is no study, to our knowledge, on human DNA depletion from positive blood cultures in BSI.

This study aimed to evaluate the feasibility and performance of nanopore sequencing with adaptive sampling for rapid pathogen identification and antimicrobial resistance prediction directly from positive blood cultures. Our results demonstrate that this approach enables accurate species identification within 1 h and comprehensive AMR profiling within 15 h, including detection of polymicrobial infections and novel species.

## Methods

### Specimen and conventional testing

Positive blood cultures were systematically collected at Taichung Veterans General Hospital and incubated in the BD BACTECTM FX system with aerobic and anaerobic BACTEC™ culture bottles. Upon microbial growth detection, blood culture broth was used for smear preparation, subcultured onto appropriate growth media. Identification used MALDI-TOF Vitek MS (bioMerieux), and susceptibility testing utilized the Vitek 2 system (bioMerieux). Following standard microbiological procedures, 1.8 ml of residual positive blood culture broth per sample was centrifuged at 13,000 g for 2 min. After removing the supernatant, the pellet was stored at 4 °C for subsequent DNA extraction and nanopore sequencing. To assess the background level of bacterial DNA contamination, six negative control blood culture bottles were included in the study.

This study included patients aged 16 and above with fever (> 38.0 °C), chills, or at least one sign of infection or systemic inflammatory response, such as a heart rate exceeding 90 beats per minute or a respiratory rate over 20 breaths per minute. This study is approved by the Institutional Review Board at Taichung Veterans General Hospital (CE22004B). A total of 458 positive blood cultures were collected over a 10-month period from patients with bloodstream infections.

### DNA extraction

The DNA extraction process followed the instructions of the QIAamp BiOstic Bacteremia DNA Kit (Qiagen GmbH, Cat No 12240–50, Hilden, Germany) on an automated QIAcube Connect machine (Qiagen GmbH, cat no 9002864, Hilden, Germany). The sample was mechanically lysed using Precellys 24 tissue homogenizer (Bertin Technologies SAS, France) at 6800 rpm bead beating for 30 s, paused for 60 s, repeated for 3 cycles.

### Library construction for nanopore sequencing

The extracted DNA was used in conjunction with the ONT Rapid barcoding kit 96 (SQK-RBK110.96) for constructing the sequencing library. DNA input mass was adjusted with nuclease-free water to a total of 400 ng in a volume of 7.5 μl then added to one of the 2.5 μl rapid barcodes (RB01-96). The DNA library was loaded onto a GridION SpotON flowcell R9.4.1 (FLO-MIN106) for sequencing. MinKNOW v22.10.7 with live base-calling and demultiplexing implemented on guppy v6.3.9 using high accuracy mode was set for at least a 15-h sequencing run. Adaptive sampling depletion mode for all channels was switched on with Human Build 38 (https://www.ncbi.nlm.nih.gov/assembly/GCF_000001405.26/) as the reference sequence.

### Bioinformatic analysis

All custom scripts utilized in the analysis were written in Python and are freely accessible in the GitHub repository: https://github.com/jade-nhri/nanoBSI (GPL-3.0 license) [27]. A reference database containing archaea, bacteria, and fungi sequences from RefSeq was established. Sequencing reads were taxonomically classified by Centrifuge 1.0.4 [28] to species level with minimum length of partial hits of 60 bp. In our pipeline, we first retrieved the top five reference genome sequences for each sample. Then, only the genome sequences covered by the sample’s reads by more than 75% were retained. Sequencing reads were de novo assembled by Flye 2.9.2 (with –meta option) to generate an assembly [29, 30]. Contigs were aligned with the retained genomes to identify covered genome sequences. Contigs were also assigned to multiple genomes based on sequencing depth, sequence identity, and circularity, if necessary. If no reference genome was chosen for a sample, the top species from Centrifuge results was used for species identification, and “L” (indicating low) was assigned for credibility. If a reference genome was selected but did not match the top species from Centrifuge results, “M” (indicating medium) credibility was assigned. High credibility (“H”) was only assigned to a sample when the selected reference matched to Centrifuge’s top species. Additionally, “C” and “IC” (signifying complete and incomplete) were appended to “H” when contig-aligned genome coverage was ≥ 90% and < 70%, respectively. Sequencing reads or species-assigned contigs were independently analyzed for AMR prediction using ResFinder 4.3.2 [31] and PointFinder [32].

### Verification of polymicrobial infections through full-length 16S rRNA sequencing

Polymicrobial-infection samples identified by nanopore sequencing underwent further processing by amplifying the V1-V9 region using primers 27F and 1492R. Subsequently, PacBio Sequel IIe system was used to produce HiFi reads with quality higher than Q30. One Codex platform (https://www.onecodex.com/) with a built-in Targeted Loci database was used for 16S analysis.

## Results

### Reference techniques uncover epidemics and AST profiles in central Taiwan

**Fig. 1:**
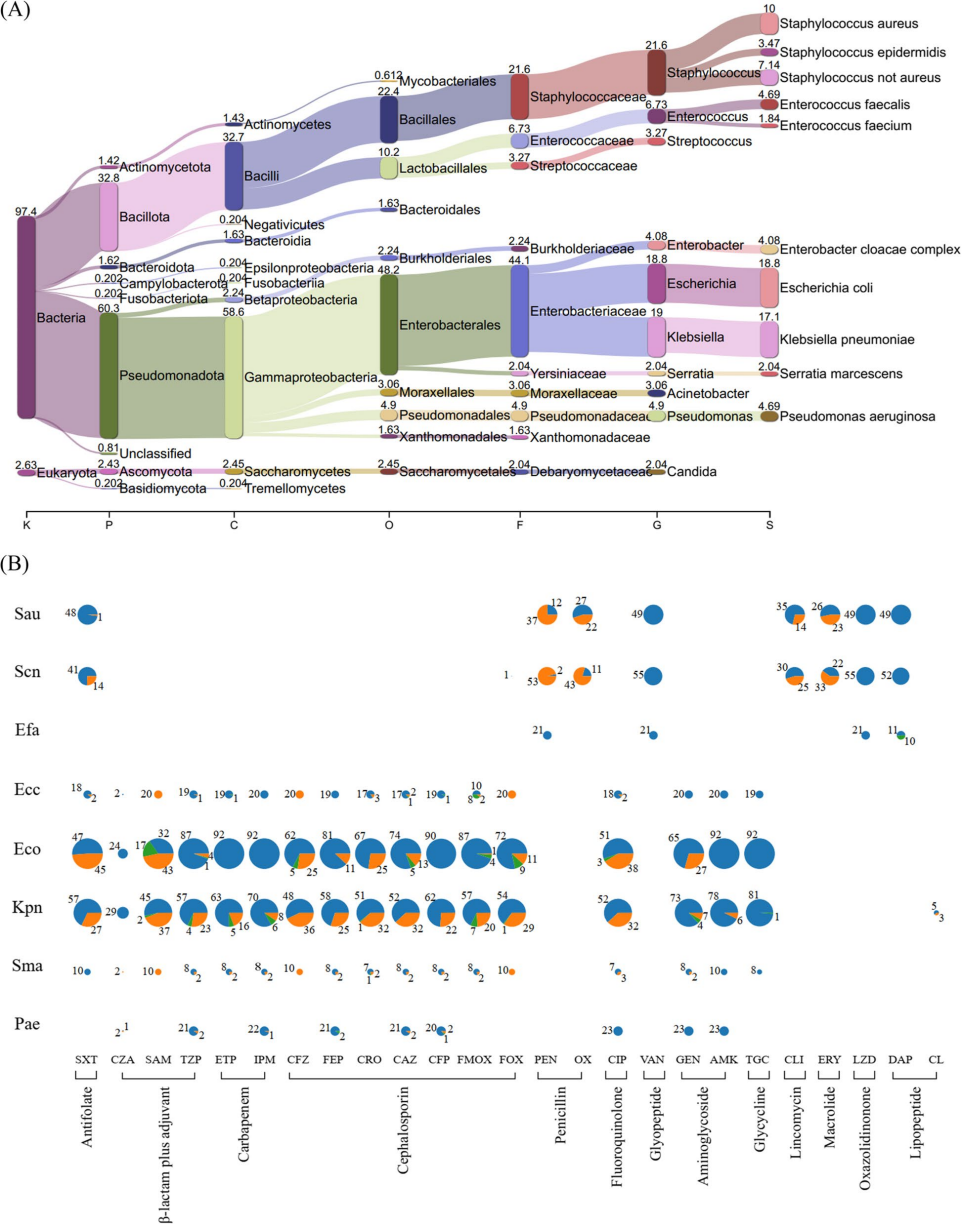
**A** Frequency distribution of the predominant pathogens responsible for bloodstream infections identified via MALDI-TOF in this study. **B** Frequency of antimicrobial susceptibility testing (AST) for various antibiotics (grouped by antibiotic class) by species. Sau, *Staphylococcus aureus*; Scn, *Staphylococcus*, coagulase negative, i.e.,* S. epidermidis*, and* S.* not *aureus* identified by MALDI-TOF; Efa, *Enterococcus faecalis*; Ecc, *Enterobacter cloacae* complex; Eco, *Escherichia coli*; Kpn, *Klebsiella pneumoniae*; Sma, *Serratia marcescens*; Pae, *Pseudomonas aeruginosa*; SXT, trimethoprim-sulfamethoxazole; CZA, ceftazidime-avibactam; SAM, ampicillin-sulbactam; TZP, piperacillin-tazobactam; ETP, ertapenem; IPM, imipenem; CFZ, cefazolin; FEP, cefepime; CRO, ceftriaxone; CAZ, ceftazidime; CFP, cefoperazone; FMOX, flomoxef; FOX, cefoxitin; PEN, penicillin; OX, oxacillin; CIP, ciprofloxacin; VAN, vancomycin; GEN, gentamicin; TGC, tigecycline; CLI, clindamycin; ERY, erythromycin; LZD, linezolid; DAP, daptomycin. CL, colistin

A total of 458 positive blood cultures were collected over a 10-month period from patients with bloodstream infections. Among these, ten samples were recorded as “No growth” upon subculture, while four samples were not characterized by MALDI-TOF, with no reported outcomes. The remaining 444 positive blood cultures grew microbial isolates, of which 400 (90%) were found to contain a single species indicating monomicrobial infection. The other 44 positive blood cultures (10%) contained two or more microbial species, indicating polymicrobial infection. A total of 494 microbial isolates identified by MALDI-TOF were collected. Fig. 1A illustrates the species composition of the isolates, highlighting that *Escherichia coli*, at 18.8%, and *Klebsiella pneumoniae*, at 17.1%, were the most frequently identified pathogens. They were followed by *Staphylococcus aureus*, accounting for 10% of the isolates, and other non-aureus *Staphylococcus* species, comprising 7.14%. Data shows roughly one third of infections were from gram-positive pathogens, including *Staphylococcus*, *Enterococcus*, and *Streptococcus* species. In contrast, approximately half were due to gram-negative pathogens, predominantly from the Enterobacterales order. Fungal infections were isolated at a rate of only 2.6% (13 out of 494), with *Candida* being the most common, accounting for 10 out of the 13 cases.

Among the total isolates, 472 underwent Vitek 2 antimicrobial susceptibility testing (AST), outlined in Additional file 1: Table S1. Fig. 1B presents the AST results for the top eight species. Notably, a significant level of antibiotic resistance was observed in *Staphylococcus* species, particularly against antibiotics such as penicillin, oxacillin, clindamycin, and erythromycin. Moreover, coagulase-negative *Staphylococcus* strains displayed a notably high resistance rate to trimethoprim-sulfamethoxazole.

In the case of *E. coli* isolates, they retained susceptibility to a range of antibiotics, including ceftazidime-avibactam, ertapenem, imipenem, amikacin, and tigecycline. On the other hand, *K. pneumoniae* isolates were fully susceptible only to ceftazidime-avibactam and tigecycline.

### Nanopore sequencing with adaptive sampling enables species identification and resistance prediction

**Fig. 2:**
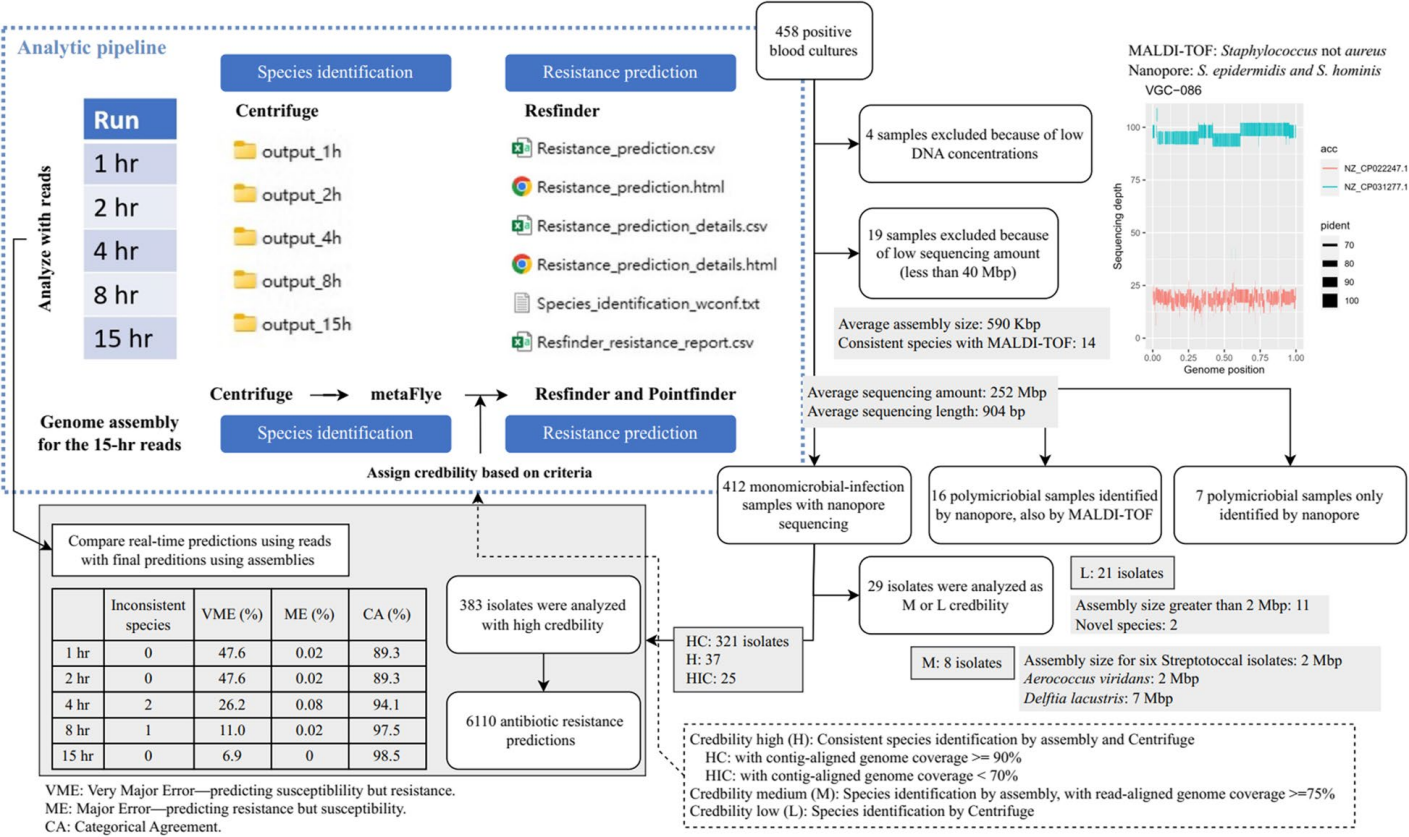
Analytic pipeline and the corresponding prediction results using nanopore sequencing with adaptive sampling

Out of the 458 positive blood cultures, four samples were excluded from nanopore sequencing due to low DNA concentrations following DNA extraction. Two of these samples were confirmed to have no growth after 4 weeks. The remaining 454 samples were processed using a total of 38 flow cells, with twelve samples pooled in each run. The 15-h sequencing reads (Additional file 1: Table S2) were analyzed to generate a total of 478 prediction results, as detailed in Additional file 1: Table S3. This process encompassed species identification and resistance prediction, executed separately with reads and genome assemblies, as depicted in Fig. 2. However, one sample failed to produce a FASTQ file at the 15-h mark. Following the exclusion of an additional 18 samples with a sequencing amount of less than 40 Mbp, the remaining 435 samples had an average sequencing amount of 252 Mbp and an average sequencing length of 904 bp (refer to Table S2). Among the nineteen low-throughput samples, two had no reported outcome, and one was recorded as “No growth” (Additional file 1: Table S4). Despite the insufficient sequencing reads (< 40 Mbp) for genome assembly, species identified using reads by Centrifuge were consistent with MALDI-TOF in 14 out of the 19 samples (Table S4). As depicted in Fig. 2, twenty-three samples were identified as polymicrobial (Table S3 and Additional file 2: Fig. S1), with 16 of them also confirmed through MALDI-TOF verification. However, seven samples were only identified as polymicrobial infections by nanopore sequencing. As shown in Fig. 2, VGC-086 was identified as *S.* not *aureus* by MALDI-TOF, but two draft genomes were assembled and separately assigned as *S. epidermidis* and *S. homini*s by nanopore sequencing. The co-infection involving these two species was further confirmed through full-length 16S rRNA sequencing, as depicted in Additional file 2: Fig. S2, confirming the presence and authenticity of the seven identified polymicrobial infections.

Among the 412 monomicrobial infections, 383 were predicted with high credibility and categorized as “H” (37 samples), “HC” (321 samples, with coverage ≥ 90%), or “HIC” (25 samples, with coverage < 70%), depending on the level of genome completeness. Although three samples showed no growth upon subculture, their sequencing reads were successfully assembled and classified as high credibility with complete genome coverage, labeled as “HC” (VGC-057, VGC-158, and VGC-306 in Additional file 1: Table S5), while one sample (VGC-277) with no reported outcome was labeled as “HIC.” The high credibility of the “HC” predictions for the three samples suggest that sampling bias or unsuitable culture conditions may have contributed to the lack of growth in subculture, potentially leading to misidentification of blood stream infections. As listed in Fig. 2, reliable species recognition was accomplished using the reads from the first hour. Only two inconsistent pairs were observed between Centrifuge (using later collected reads) and the reference assignment to genome assembly: *Bacillus pumilus* vs. *Bacillus altitudinis* and *Enterobacter cloacae complex sp.* vs. *Enterobacter hormaechei*. Although a considerably high categorical agreement (94.1%) could be obtained using the 4-h reads compared to the final prediction, the substantial very major error rate (26.2%) suggests that collecting more reads is inevitable for resistant gene detection. In addition to the high credibility, 29 isolates were classified as medium (8 isolates) and low credibility (21 isolates). While genome assemblies and their corresponding reference genomes were obtained for the 8 samples, the species identified in reference genomes did not match with the species assignments made using Centrifuge (see Table S4 for details). Six out of the eight samples were all recognized as Streptococcal isolates, but differing species assignments resulted from the different approaches—MALDI-TOF, Centrifuge and assembly. The other predictions of medium credibility were *Aerococcus viridans* and *Delftia lacustris*; the former aligned with the MALDI-TOF result, while the latter had no corresponding report. The other 21 samples were labeled as “L” because we could not find reference genomes for mapping with assemblies. Additionally, based on the background level of bacterial contamination in negative controls, with an average classified rate of 1.9%, and an SD of 0.17%, a threshold for pathogen detection was established. Classified reads below 2.4% (average + 3 × SD) were considered "No detection". Nine samples were thus categorized as “No detection,” and four of these were found to have “No growth.” Nevertheless, we successfully obtained 11 genome assemblies with size exceeding 2 Mbp, and two of them were likely to be novel species (assembly statistic in Table S4).

### Species identification through nanopore sequencing provides enhanced resolution

**Fig. 3:**
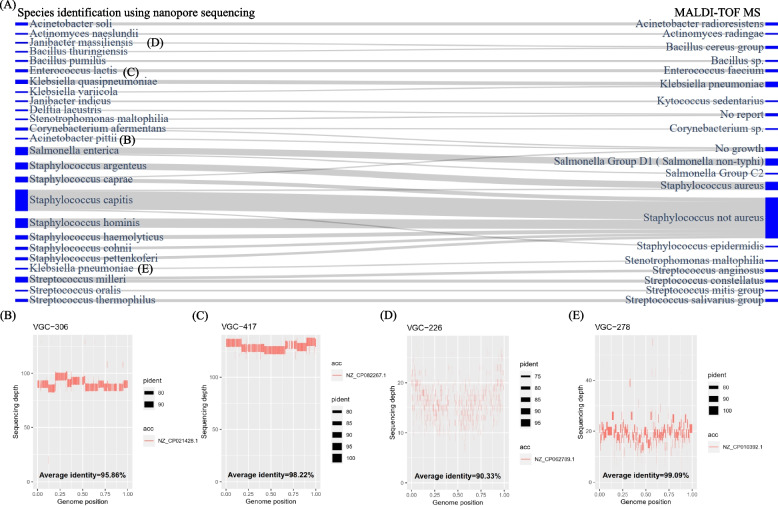
**A** Inconsistent pairs of species identification between nanopore sequencing and MALDI-TOF. **B**–**E** Genome coverages aligned with contigs and their average identities to reference genomes. Please note that the sequencing depth information was extracted from assembly_info.txt generated by Flye

To conduct a parallel comparison between MALDI-TOF and nanopore sequencing, a subset of 387 monomicrobial-infection samples (Additional file 2: Fig. S3) was used. Among them, 303 showed consistent results (Table S5), but 84 instances were inconsistent. To exclude 11 low credibility samples, 73 isolates underwent further comparative analysis. As depicted in Fig. 3A, several instances of species identified under the same genus were noted, such as *K. pneumoniae* to *K. quasipneumoniae*, *Salmonella* non-typhi to *S. enterica*, and *S.* not *aureus* to *S. capitis* or *S. hominis*. Figs. 3B and 3C show the contig-aligned genome coverages and average identities for reference genomes (*Acinetobacter pittii* (NZ_CP021428) and *Enterococcus lactis* (NZ_CP082267)), indicating complete and reliable draft assemblies for isolates VGC-306 and VGC-417, but they were reported as “No growth” and *E. faecium*, respectively, by MALDI-TOF. Based on the species-specific primers designed in the study to accurately differentiate between *E. lactis* and *E. faecium* [33], we have validated in silico that VGC-417 is *E. lactis*. Two particularly noteworthy inconsistent pairs, VGC-226 (*Janibacter massiliensis* vs. *Bacillus cereus*) and VGC-278 (*Klebsiella pneumoniae* vs. *Stenotrophomonas maltophilia*), are illustrated in Figs. 3D and 3E. Despite fragmented draft genomes, they exhibited complete contig-aligned genome coverages. The high identity percentage (99%) for VGC-278 confidently assigns it to *K. pneumoniae*. Conversely, VGC-226 is suggested to be a potential novel *Janibacter* species, given its highest known reference genome identity is only 90%. The charts depicting contig-aligned genome coverage plot and average identity for the 73 isolates can be found in Additional file 2: Fig. S4.

### A modified ResFinder database allows accurate susceptibility predictions

From 387 monomicrobial infection samples, 4195 AST results were obtained, excluding 90 intermediates. Original ResFinder and PointFinder were used for AMR prediction but resulted in a categorical agreement rate of 85.9% and a very major error (VME) rate of 35.8% (Table 1). A significant discrepancy was noted in the case of *S. aureus*, particularly related to oxacillin resistance. Two thirds of *S. aureus* cases were linked to oxacillin, but the *mecA* gene was absent in the original ResFinder database. Additionally, the database lacked genetic determinants for cefazolin resistance, leading to 69 very major errors (detailed in Additional file 1: Table S6). To address these issues, we curated a list of gene-antibiotic associations (Additional file 1: Table S7) to update the ResFinder database. This update also incorporated information about inherent antibiotic resistance in specific bacterial species (Additional file 1: Table S8). Therefore, the overall categorical agreement rate improved significantly, exceeding 90%, and the VME rate decreased dramatically from 35.8 to 10.1% (Table 1). Remarkably, the study achieved very low VME rates for key pathogens: 1.1% for *Klebsiella pneumoniae*, 1.1% for *Staphylococcus aureus*, and 2.6% for *Escherichia coli*. The VME rates for *K. pneumoniae* and *S. aureus* were below 1.5%, meeting the FDA’s requirements for diagnostic tests. These results highlight the potential of nanopore sequencing for accurate antibiotic susceptibility predictions.

**Table 1:** Evaluation of antimicrobial resistance prediction performance

			Original ResFinder			Modified ResFinder		
	No. of isolates	No. of AST	CA	VME	ME	CA	VME	ME
Monomicrobial infections	387	4195	85.9	35.8	8.2	90.6	10.1	9.2
HC (high credibility with complete assembly)	305	3442	86.4	33.7	8.0	91.4	7.2	9.0
HC: *Escherichia coli*	59	931	92.8	15.7	5.5	94.1	2.6	6.6
HC: *Klebsiella pneumoniae*	40	634	81.1	29.6	14.3	89.1	**1.1**	14.8
HC: *Staphylococcus aureus*	41	369	91.6	33.3	0.4	97.3	**1.1**	3.2
HC: *Staphylococcus*, coagulase negative	55	482	87.6	37.8	1.8	95.0	10.5	2.7
H	35	419	83.1	35.4	13.6	86.6	4.6	15.0
HIC, M, and L	47	334	80.6	56.5	3.6	87.1	42.9	3.9

*CA* categorical agreement (%), *VME* very major error (%), false susceptibility—predicting susceptibility but actual resistance by Vitek 2, *ME* major error (%), false resistance—predicting resistance but actual susceptibility by Vitek 2

Bold entries denote VME values below 1.5%, meeting the requirement set by the Food and Drug Agency (FDA)

## Discussion

In this study, we employed nanopore sequencing with human DNA depletion mode to generate genomic information on the causative agent in bloodstream infections. We found that reliable species identification can be obtained using the sequencing reads generated within the first hour, and the draft genomes assembled from the 15-h reads can be complete and novel. Examples include the assemblies of VGC-001, VGC-021, and VGC-022 (see Table S5), which had 6, 4, and 5 contigs, with genome sizes of 2.5, 5.4, and 2.8 Mbp for *S. capitis*, *K. pneumoniae*, and *S. aureus*, respectively. While VGC-035 and VGC-229 were assigned by our analytic pipeline with low credibility, their genomes were assembled into 10 and 4 contigs, respectively. These two assemblies were classified by MiGA [34] to the genus *Microbacterium* with *p*-value of 0.01 and the family Cellulomondaceae with *p*-value of 0.0058, suggesting they represent novel species. By utilizing a database-assisted species assignment, a total of 76 distinct species were comprehensively identified from over 450 bloodstream infection samples (Table S3), including fungal pathogens such as *Candida albicans*, *Candida glabrata*,* Candida parapsilosis*,* Candida tropicalis*, and *Cryptococcus neoformans*. Besides, the draft assemblies elucidated the connections between antimicrobial resistance phenotype and genotype (Additional file 2: Fig. S5-S8).

The advent of nanopore sequencing with adaptive sampling in BSI diagnostics, particularly for the high incidence of methicillin-resistant *Staphylococcus aureus* (MRSA) [35, 36], heralds a significant improvement in predicting AMR and guiding timely therapeutic interventions. Studies have consistently demonstrated the critical importance of timely administration of appropriate antimicrobials in patients with bacteremia. For instance, in community-onset bacteremia, the optimal timing for administering effective antimicrobials is within the first 48 h of arrival at the emergency department [37]. Additionally, the delay in initiating antimicrobial therapy in MRSA bacteremia is associated with a higher mortality rate compared to susceptible *S. aureus* [36, 38]. In this context, our approach can accurately predict MRSA 48 h earlier than conventional methods. This accelerated and precise identification of AMR enables clinicians to initiate appropriate antimicrobial therapy much sooner, which is critical in reducing mortality and morbidity associated with MRSA bacteremia. The capacity to rapidly and accurately determine the AMR profile of the pathogen significantly enhances the effectiveness of the chosen therapeutic regimen, aligning with the findings that timely and appropriate antimicrobial administration is a primary determinant of survival in BSI patients. Therefore, the integration of nanopore sequencing into clinical practice represents a transformative step in the management of BSI, offering a more timely, precise, and effective approach to antimicrobial stewardship and improving patient outcomes in cases of MRSA and other serious infections [39].

While our nanopore sequencing approach offers comprehensive pathogen identification and AMR prediction, it is important to compare its performance with other rapid AST devices, such as the FDA-cleared Accelerate PhenoTest BC system [8, 9, 40] and the Q-linea ASTar system [11, 12]. These systems deliver results relative quickly, with the Accelerate PhenoTest providing organism identification in 1.5–6.9 h and AST results in 5.8–16.5 h [40]. Although this is somewhat faster than our current nanopore sequencing protocol, which requires 15 h for full AMR prediction, we can still provide initial species identification within 1 h. It is import to note, however, that these phenotypic systems are limited to pre-defined panels of bacterial species and antibiotics, primarily focusing on gram-negative bacteria [12]. The Accelerate PhenoTest, for example, covers 6 gram-positive and 8 gram-negative bacteria, along with two *Candida* species. In contrast, our nanopore sequencing approach enables the identification of a broader range of pathogens, including novel species, with 76 distinct species identified in this study.

In terms of AST accuracy, the ASTar system reports categorical agreements of 95.6–97.6% with a very major error rate of 2.4% [11, 12], while the Accelerate PhenoTest shows a category agreement of 96.4% with a 1.0% very major discrepancy rate [41]. Our optimized method achieved an overall categorical agreement of 90.6%, with very low VME rates for key pathogens such as *K. pneumoniae* (1.1%) and *S. aureus* (1.1%), approaching the performance of these specialized systems. Furthermore, our method provides valuable genomic information that can be utilized for outbreak investigations, virulence profiling, and tracking the evolution of AMR—capabilities that phenotypic AST devices do not offer. Additionally, we have demonstrated the ability to detect polymicrobial infections, which can be challenging for phenotypic systems.

The integration of nanopore sequencing into routine clinical workflows for BSI diagnostics holds significant promise for transforming patient care. However, successful implementation requires careful consideration of several practical factors. While the initial investment for nanopore sequencing platforms is relatively low, ongoing costs, including consumables such as flow cells ($700 each), library preparation kits (Rapid Barcoding Kit at $100 per run), and DNA extraction ($5 per sample), as well as maintenance expenses, should be considered in budget planning. Fortunately, the cost of sequencing continues to decrease (from approximate $75 to $50 per sample when purchasing 96 flow cells in bulk), making it increasingly accessible to clinical laboratories. Laboratory personnel will require specialized training in various aspects of nanopore sequencing, including DNA extraction, library preparation, sequencing operation, and bioinformatic analysis. The increasing availability of user-friendly protocols, software, and training programs from ONT is facilitating this training process. For routine clinical diagnostic use, regulatory approval (e.g., FDA clearance) is essential. This necessitates rigorous validation studies to demonstrate the accuracy, reliability, and clinical utility of the technology. As more real-world data emerge and standardized protocols are established, obtaining regulatory approval will become more feasible.

As nanopore sequencing technology matures, costs decrease, and user-friendly tools become more readily available, the barriers to clinical integration will diminish. Swiftly identifying pathogens and their resistance profiles can contribute to more targeted antimicrobial therapies, potentially shortening hospital stays, mitigating complication risks, and ultimately reducing overall healthcare expenses. Further investigations are warranted to quantify these potential economic benefits.

This study has several limitations. First, it is limited to a single-hospital design, and the AST results rely exclusively on the Vitek 2 system. Second, we restricted our analyses to species identification and antibiotic resistance predictions. It would be valuable to discriminate isolates at the genomic subtype level for exploring epidemic trends or to identify genotypic determinants of virulence [16]. Additionally, virulence and stress response factors can be identified using AMRFinderPlus (Additional file 1: Table S9) [42]. Alternatively, tracking plasmid backbones of concern [43] could also be pursued using the draft assemblies obtained in this study. Third, as the mechanisms mediating antimicrobial resistance can vary among bacterial genera, distinct antimicrobial determinant databases are necessary [13, 42]. Although we have introduced a modified ResFinder database, continuous updates are still necessary to identify and integrate newly discovered genes and mutations associated with antimicrobial resistance.

In summary, nanopore sequencing technology enables the direct and precise identification of pathogens and the prediction of antimicrobial resistance from positive blood cultures within a 24-h timeframe. Our system delivers timely and informative results, ultimately enhancing outcomes for patients with bloodstream infections.

## Conclusions

This study demonstrates that nanopore sequencing with adaptive sampling offers rapid and comprehensive pathogen identification and AMR prediction directly from positive blood cultures, enabling results within a clinically relevant timeframe. This approach has the potential to significantly improve bloodstream infection management by facilitating earlier targeted therapy and informing more effective antimicrobial stewardship strategies.

## Supplementary Information

Additional file 1: Supplementary Table S1-Table S9.

Additional file 2: Supplementary Fig. S1-S8.

## Data Availability

The sequencing data generated during the current study are available in the Sequence Read Archive (SRA) under accession number PRJNA1039556 (https://www.ncbi.nlm.nih.gov/sra/?term=PRJNA1039556) [44].

## References

[1] Laupland KB. Incidence of bloodstream infection: a review of population-based studies. Clin Microbiol Infect. 2013;19:492–500. 23398633 10.1111/1469-0691.12144

[2] Timsit JF, Ruppe E, Barbier F, Tabah A, Bassetti M. Bloodstream infections in critically ill patients: an expert statement. Intensive Care Med. 2020;46:266–84. 32047941 10.1007/s00134-020-05950-6 PMC7223992

[3] Kontula KSK, Skogberg K, Ollgren J, Jarvinen A, Lyytikainen O. Population-based study of bloodstream infection incidence and mortality rates, Finland, 2004–2018. Emerg Infect Dis. 2021;27:2560–9. 34546161 10.3201/eid2710.204826 PMC8462341

[4] Schoneweck F, Schmitz RPH, Rissner F, Scherag A, Loffler B, Pletz MW, Weis S, Brunkhorst FM, Hagel S. The epidemiology of bloodstream infections and antimicrobial susceptibility patterns in Thuringia, Germany: a five-year prospective, state-wide surveillance study (AlertsNet). Antimicrob Resist Infect Control. 2021;10:132. 34493334 10.1186/s13756-021-00997-6 PMC8424790

[5] Rodrigues Pires de Campos L, Farrel Cortes M, Deo B, Rizek C, Santos S, Perdigao L, Costa SF. Risk factors for bloodstream infection by multidrug-resistant organisms in critically ill patients in a reference trauma hospital. Am J Infect Control. 2022;50:673–9. 10.1016/j.ajic.2021.10.020 34756966

[6] Rydzak T, Groves RA, Zhang R, Aburashed R, Pushpker R, Mapar M, Lewis IA. Metabolic preference assay for rapid diagnosis of bloodstream infections. Nat Commun. 2022;13:2332. 35484129 10.1038/s41467-022-30048-6 PMC9050716

[7] Zalas-Wiecek P, Bogiel T, Gospodarek-Komkowska E. The accelerate pheno system-a new tool in microbiological diagnostics of bloodstream infections: a pilot study from Poland. Pathogens. 2022;11:1415. 10.3390/pathogens11121415 PMC9781321 36558749

[8] Brosh-Nissimov T, Tzur A, Grupel D, Cahan A, Ma’aravi N, Heled-Akiva M, Jawamis H, Leskes H, Barenboim E, Sorek N. Clinical impact of the accelerate PhenoTest(R) BC system on patients with gram-negative bacteremia and high risk of antimicrobial resistance: a prospective before-after implementation study. Ann Clin Microbiol Antimicrob. 2023;22:62. 37516885 10.1186/s12941-023-00619-6 PMC10387206

[9] Banerjee R, Komarow L, Virk A, Rajapakse N, Schuetz AN, Dylla B, Earley M, Lok J, Kohner P, Ihde S, et al. Randomized trial evaluating clinical impact of RAPid identification and susceptibility testing for gram-negative bacteremia: RAPIDS-GN. Clin Infect Dis. 2021;73:e39–46. 32374822 10.1093/cid/ciaa528 PMC8246790

[10] Bhalodi AA, MacVane SH, Ford B, Ince D, Kinn PM, Percival KM, Bremmer DN, Carr DR, Walsh TL, Bhatti MM, et al. Real-world impact of the Accelerate PhenoTest BC Kit on patients with bloodstream infections in the improving outcomes and antimicrobial stewardship study: a quasiexperimental multicenter study. Clin Infect Dis. 2022;75:269–77. 34718456 10.1093/cid/ciab921 PMC9410719

[11] Esse J, Trager J, Valenza G, Bogdan C, Held J. Rapid phenotypic antimicrobial susceptibility testing of Gram-negative rods directly from positive blood cultures using the novel Q-linea ASTar system. J Clin Microbiol. 2023;61:e0054923. 10.1128/jcm.00549-23 PMC10662367 37819072

[12] Goransson J, Sundqvist M, Ghaderi E, Lisby JG, Molin Y, Eriksson E, Carlsson S, Cederlof A, Ellis L, Melin J. Performance of a system for rapid phenotypic antimicrobial susceptibility testing of gram-negative bacteria directly from positive blood culture bottles. J Clin Microbiol. 2023;61: e0152522. 36852983 10.1128/jcm.01525-22 PMC10035315

[13] Baker KS, Jauneikaite E, Hopkins KL, Lo SW, Sanchez-Buso L, Getino M, Howden BP, Holt KE, Musila LA, Hendriksen RS, et al. Genomics for public health and international surveillance of antimicrobial resistance. Lancet Microbe. 2023;4:e1047–55. 10.1016/S2666-5247(23)00283-5 37977162

[14] Baker KS, Jauneikaite E, Nunn JG, Midega JT, Atun R, Holt KE, Walia K, Howden BP, Tate H, Okeke IN, et al. Evidence review and recommendations for the implementation of genomics for antimicrobial resistance surveillance: reports from an international expert group. Lancet Microbe*.* 2023;4:e1035–9. 10.1016/S2666-5247(23)00281-1 37977164

[15] Hall MB, Rabodoarivelo MS, Koch A, Dippenaar A, George S, Grobbelaar M, Warren R, Walker TM, Cox H, Gagneux S, et al. Evaluation of Nanopore sequencing for *Mycobacterium tuberculosis* drug susceptibility testing and outbreak investigation: a genomic analysis. Lancet Microbe. 2023;4:e84–92. 36549315 10.1016/S2666-5247(22)00301-9 PMC9892011

[16] Jauneikaite E, Baker KS, Nunn JG, Midega JT, Hsu LY, Singh SR, Halpin AL, Hopkins KL, Price JR, Srikantiah P, et al. Genomics for antimicrobial resistance surveillance to support infection prevention and control in health-care facilities. Lancet Microbe. 2023;4:e1040–6. 10.1016/S2666-5247(23)00282-3 37977161

[17] Muloi DM, Jauneikaite E, Anjum MF, Essack SY, Singleton DA, Kasudi MR, Wade MJ, Egyir B, Nunn JG, Midega JT, et al. Exploiting genomics for antimicrobial resistance surveillance at One Health interfaces. Lancet Microbe. 2023;4:e1056–62. 10.1016/S2666-5247(23)00284-7 37977165

[18] Farzana R, Jones LS, Rahman MA, Sands K, van Tonder AJ, Portal E, Criollo JM, Parkhill J, Guest MF, Watkins WJ, et al. Genomic Insights into the mechanism of carbapenem resistance dissemination in Enterobacterales from a tertiary public heath setting in South Asia. Clin Infect Dis. 2023;76:119–33. 35412593 10.1093/cid/ciac287 PMC9825829

[19] Zhang Y, Lu X, Tang LV, Xia L, Hu Y. Nanopore-targeted sequencing improves the diagnosis and treatment of patients with serious infections. mBio. 2023*;*14:e0305522. 10.1128/mbio.03055-22 PMC9979620 36651731

[20] Whittle E, Yonkus JA, Jeraldo P, Alva-Ruiz R, Nelson H, Kendrick ML, Grys TE, Patel R, Truty MJ, Chia N. Optimizing nanopore sequencing for rapid detection of microbial species and antimicrobial resistance in patients at risk of surgical site infections. mSphere. 2022;7:e0096421. 10.1128/msphere.00964-21 PMC8849348 35171692

[21] Liu Y, Xu Y, Xu X, Chen X, Chen H, Zhang J, Ma J, Zhang W, Zhang R. CHEN J. Metagenomic identification of pathogens and antimicrobial-resistant genes in bacterial positive blood cultures by nanopore sequencing. Front Cell Infect Microbiol. 2023;13:1283094. 10.3389/fcimb.2023.1283094 PMC10773726 38192400

[22] Martin S, Heavens D, Lan Y, Horsfield S, Clark MD, Leggett RM. Nanopore adaptive sampling: a tool for enrichment of low abundance species in metagenomic samples. Genome Biol. 2022;23:11. 35067223 10.1186/s13059-021-02582-x PMC8785595

[23] Loose M, Malla S, Stout M. Real-time selective sequencing using nanopore technology. Nat Methods. 2016;13:751–4. 27454285 10.1038/nmeth.3930 PMC5008457

[24] Marquet M, Zollkau J, Pastuschek J, Viehweger A, Schleussner E, Makarewicz O, Pletz MW, Ehricht R, Brandt C. Evaluation of microbiome enrichment and host DNA depletion in human vaginal samples using Oxford Nanopore’s adaptive sequencing. Sci Rep. 2022;12:4000. 35256725 10.1038/s41598-022-08003-8 PMC8901746

[25] Lin Y, Dai Y, Zhang S, Guo H, Yang L, Li J, Wang K, Ni M, Hu Z, Jia L, et al. Application of nanopore adaptive sequencing in pathogen detection of a patient with *Chlamydia psittaci* infection. Front Cell Infect Microbiol. 2023;13:1064317. 36756615 10.3389/fcimb.2023.1064317 PMC9900021

[26] Gan M, Wu B, Yan G, Li G, Sun L, Lu G, Zhou W. Combined nanopore adaptive sequencing and enzyme-based host depletion efficiently enriched microbial sequences and identified missing respiratory pathogens. BMC Genomics. 2021;22:732. 34627155 10.1186/s12864-021-08023-0 PMC8501638

[27] Liao Y-C: nanoBSI: Bioinformatic pipeline for analyzing nanopore sequencing data from positive blood cultures in bloodstream infections. https://github.com/jade-nhri/nanoBSI: Github; 2024.

[28] Kim D, Song L, Breitwieser FP, Salzberg SL. Centrifuge: rapid and sensitive classification of metagenomic sequences. Genome Res. 2016;26:1721–9. 27852649 10.1101/gr.210641.116 PMC5131823

[29] Liao Y-C, Cheng H-W, Wu H-C, Kuo S-C. Lauderdale T-LY, Chen F-J: Completing circular bacterial genomes with assembly complexity by using a sampling strategy from a single MinION run with barcoding. Front Microbiol. 2019;10:2068. 31551994 10.3389/fmicb.2019.02068 PMC6737777

[30] Kolmogorov M, Bickhart DM, Behsaz B, Gurevich A, Rayko M, Shin SB, Kuhn K, Yuan J, Polevikov E, Smith TPL, Pevzner PA. metaFlye: scalable long-read metagenome assembly using repeat graphs. Nat Methods. 2020;17:1103–10. 33020656 10.1038/s41592-020-00971-x PMC10699202

[31] Florensa AF, Kaas RS, Clausen P, Aytan-Aktug D, Aarestrup FM. ResFinder - an open online resource for identification of antimicrobial resistance genes in next-generation sequencing data and prediction of phenotypes from genotypes. Microb Genom. 2022;8:000748. 10.1099/mgen.0.000748 PMC8914360 35072601

[32] Zankari E, Allesoe R, Joensen KG, Cavaco LM, Lund O, Aarestrup FM. PointFinder: a novel web tool for WGS-based detection of antimicrobial resistance associated with chromosomal point mutations in bacterial pathogens. J Antimicrob Chemother. 2017;72:2764–8. 29091202 10.1093/jac/dkx217 PMC5890747

[33] Belloso Daza Mireya V, Almeida-Santos Ana C, Novais C, Read A, Alves V, Cocconcelli Pier S, Freitas Ana R, Peixe L. Distinction between *Enterococcus faecium* and *Enterococcus lactis* by a gluP PCR-based assay for accurate identification and diagnostics. Microbiology Spectrum. 2022;10:e03268–03222. 10.1128/spectrum.03268-22 PMC9769498 36453910

[34] Rodriguez RL, Gunturu S, Harvey WT, Rossello-Mora R, Tiedje JM, Cole JR, Konstantinidis KT. The Microbial Genomes Atlas (MiGA) webserver: taxonomic and gene diversity analysis of Archaea and Bacteria at the whole genome level. Nucleic Acids Res. 2018;46:W282–8. 29905870 10.1093/nar/gky467 PMC6031002

[35] Ohnuma T, Chihara S, Costin B, Treggiari MM, Bartz RR, Raghunathan K, Krishnamoorthy V. Association of appropriate empirical antimicrobial therapy with in-hospital mortality in patients with bloodstream infections in the US. JAMA Netw Open. 2023;6: e2249353. 36598788 10.1001/jamanetworkopen.2022.49353 PMC9857618

[36] Aratani T, Tsukamoto H, Higashi T, Kodawara T, Yano R, Hida Y, Iwasaki H, Goto N. Association of methicillin resistance with mortality of hospital-acquired *Staphylococcus aureus* bacteremia. J Int Med Res. 2021;49:3000605211058872. 34826374 10.1177/03000605211058872 PMC8647257

[37] Lee CC, Lee CH, Hong MY, Tang HJ, Ko WC. Timing of appropriate empirical antimicrobial administration and outcome of adults with community-onset bacteremia. Crit Care. 2017;21:119. 28545484 10.1186/s13054-017-1696-z PMC5445436

[38] Melzer M, Eykyn SJ, Gransden WR, Chinn S. Is methicillin-resistant *Staphylococcus aureus* more virulent than methicillin-susceptible S. aureus? A comparative cohort study of British patients with nosocomial infection and bacteremia. Clin Infect Dis. 2003;*3*7:1453–60. 10.1086/379321 14614667

[39] Fidalgo B, Morata L, Cardozo C, Del Rio A, Morales J, Fernandez-Pittol M, Martinez JA, Mensa J, Vila J, Soriano A, Casals-Pascual C. Information delay of significant bloodstream isolates and patient mortality: a retrospective analysis of 6225 adult patients with bloodstream infections. Clin Infect Dis. 2023;77:680–6. 37099685 10.1093/cid/ciad243

[40] Stokes W, Campbell L, Pitout J, Conly J, Church D, Gregson D. Comparison of Accelerate PhenoTest BC Kit and MALDI-TOF MS/VITEK 2 System for the rapid identification and antimicrobial susceptibility testing of gram-negative bacilli causing bloodstream infections. J Assoc Med Microbiol Infect Dis Can. 2020;5:145–57. 36341310 10.3138/jammi-2020-0004 PMC9608732

[41] Marschal M, Bachmaier J, Autenrieth I, Oberhettinger P, Willmann M, Peter S. Evaluation of the Accelerate Pheno system for fast identification and antimicrobial susceptibility testing from positive blood cultures in bloodstream infections caused by gram-negative pathogens. J Clin Microbiol. 2017;55:2116–26. 28446572 10.1128/JCM.00181-17 PMC5483913

[42] Feldgarden M, Brover V, Gonzalez-Escalona N, Frye JG, Haendiges J, Haft DH, Hoffmann M, Pettengill JB, Prasad AB, Tillman GE, et al. AMRFinderPlus and the Reference Gene Catalog facilitate examination of the genomic links among antimicrobial resistance, stress response, and virulence. Sci Rep. 2021;11:12728. 34135355 10.1038/s41598-021-91456-0 PMC8208984

[43] Wheeler NE, Price V, Cunningham-Oakes E, Tsang KK, Nunn JG, Midega JT, Anjum MF, Wade MJ, Feasey NA, Peacock SJ, et al. Innovations in genomic antimicrobial resistance surveillance. Lancet Microbe. 2023;4:e1063–70. 10.1016/S2666-5247(23)00285-9 37977163

[44] Po-Yu Liu H-CW, Ying-Lan Li, Hung-Wei Cheng, Ci-Hong Liou, Feng-Jui Chen, Yu-Chieh Liao. Comprehensive pathogen identification and antimicrobial resistance prediction from positive blood cultures using nanopore sequencing technology. BioProject PRJNA1039556, NCBI Sequence Read Archive. 2024. 10.1186/s13073-024-01416-2 PMC11610257 39617907

