# Supplementary material for: Comprehensive pathogen identification and antimicrobial resistance prediction from positive blood cultures using nanopore sequencing technology
Source: Genome Med. 2024 Dec 2;16:141. doi: 10.1186/s13073-024-01416-2 (PMC11610257; doi:10.1186/s13073-024-01416-2)
Supplement: Supplementary file 2 — Additional file 2: Supplementary Fig. S1-S8. [file 13073_2024_1416_MOESM2_ESM.pdf]

Table of Contents

Fig. S1: Genome coverages aligned with contigs and their average identities to reference genomes from polymicrobial samples 2

Fig. S2: Confirmation of polymicrobial infections through full-length 16S rRNA sequencing 6

Fig. S3: Schematic workflow of the study 16

Fig. S4: Genome coverages aligned with contigs and their average identities to reference genomes from the 73 inconsistent pairs of species identification between nanopore sequencing and MALDI-TOF 17

Fig. S5: Correlation between antimicrobial resistance phenotypes and genotypes in *Escherichia coli* 30

Fig. S6: Correlation between antimicrobial resistance phenotypes and genotypes in *Klebsiella pneumoniae* 31

Fig. S7: Correlation between antimicrobial resistance phenotypes and genotypes in *Staphylococcus aureus* 32

Fig. S8: Correlation between antimicrobial resistance phenotypes and genotypes in *Staphylococcus*, coagulase negative 33

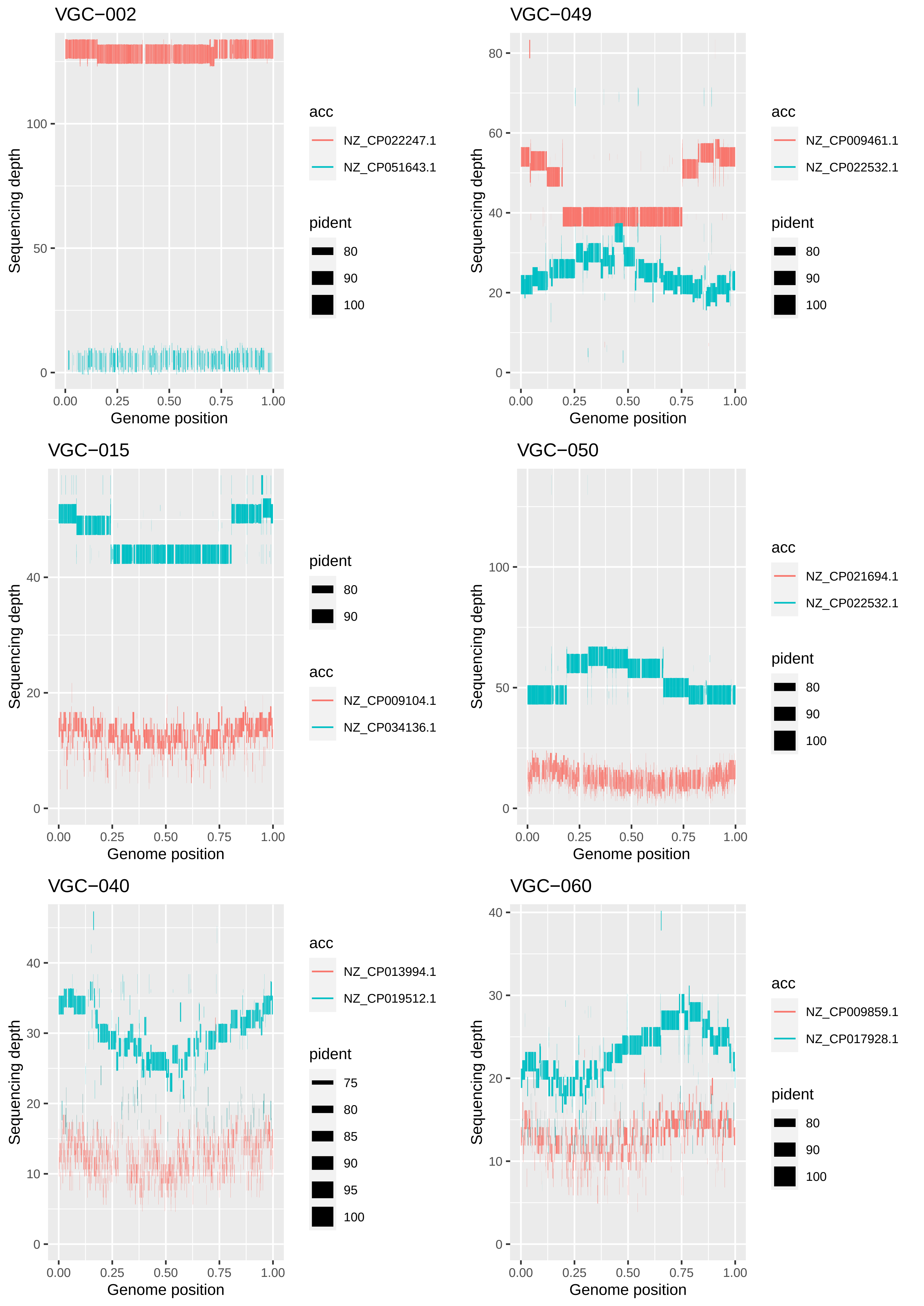
VGC−002
Sequencing depth
Genome position
acc
NZ_CP022247.1
NZ_CP051643.1
pident
80
90
100
VGC−049
acc
NZ_CP009461.1
NZ_CP022532.1
pident
80
90
100
VGC−015
pident
80
90
acc
NZ_CP009104.1
NZ_CP034136.1
VGC−050
acc
NZ_CP021694.1
NZ_CP022532.1
pident
80
90
100
VGC−040
acc
NZ_CP013994.1
NZ_CP019512.1
pident
75
80
85
90
95
100
VGC−060
acc
NZ_CP009859.1
NZ_CP017928.1
pident
80
90
100

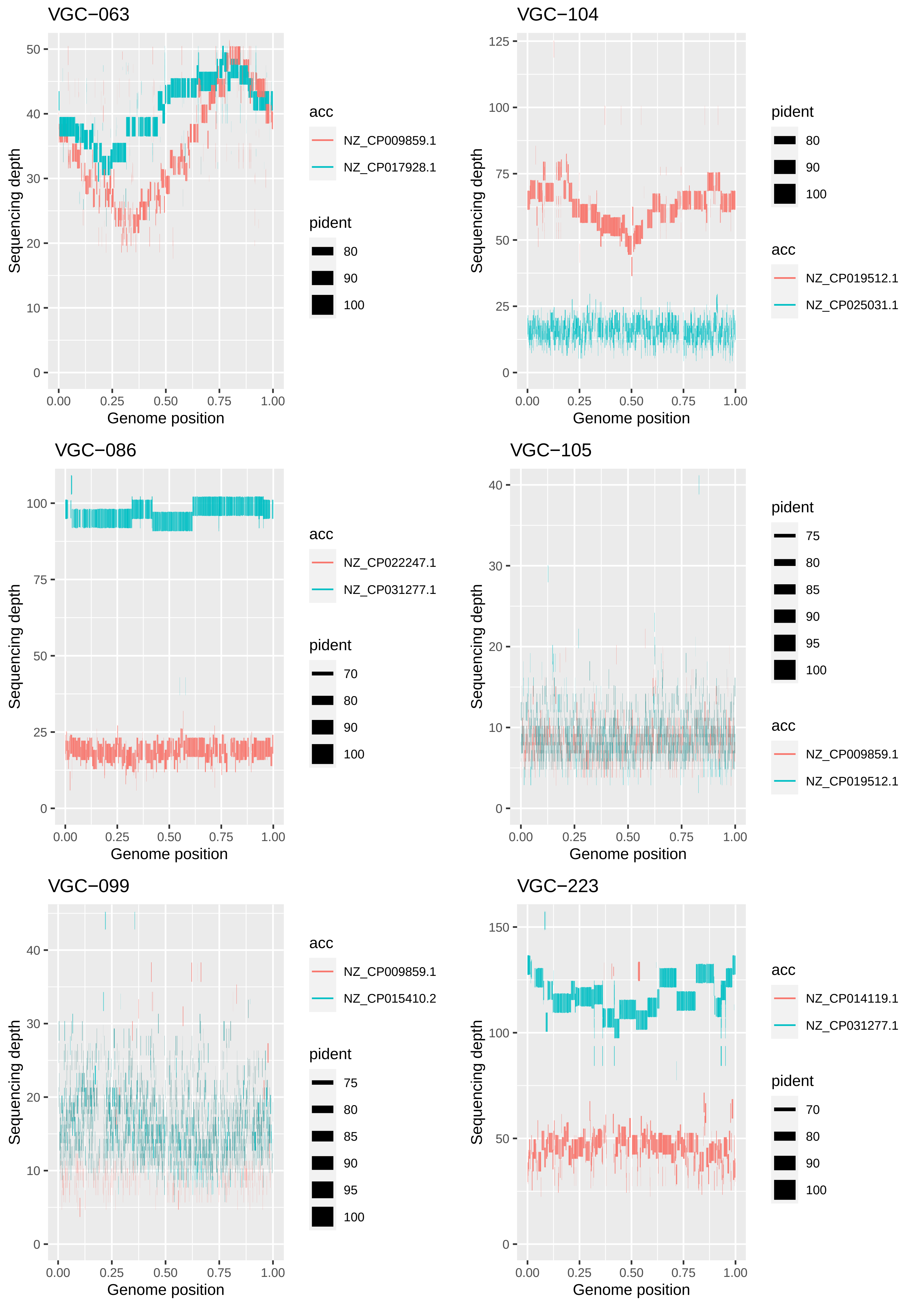
VGC−063
Sequencing depth
Genome position
acc
NZ_CP009859.1
NZ_CP017928.1
pident
80
90
100
VGC−104
pident
80
90
100
acc
NZ_CP019512.1
NZ_CP025031.1
VGC−086
acc
NZ_CP022247.1
NZ_CP031277.1
pident
70
80
90
100
VGC−105
pident
75
80
85
90
95
100
acc
NZ_CP009859.1
NZ_CP019512.1
VGC−099
acc
NZ_CP009859.1
NZ_CP015410.2
pident
75
80
85
90
95
100
VGC−223
acc
NZ_CP014119.1
NZ_CP031277.1
pident
70
80
90
100

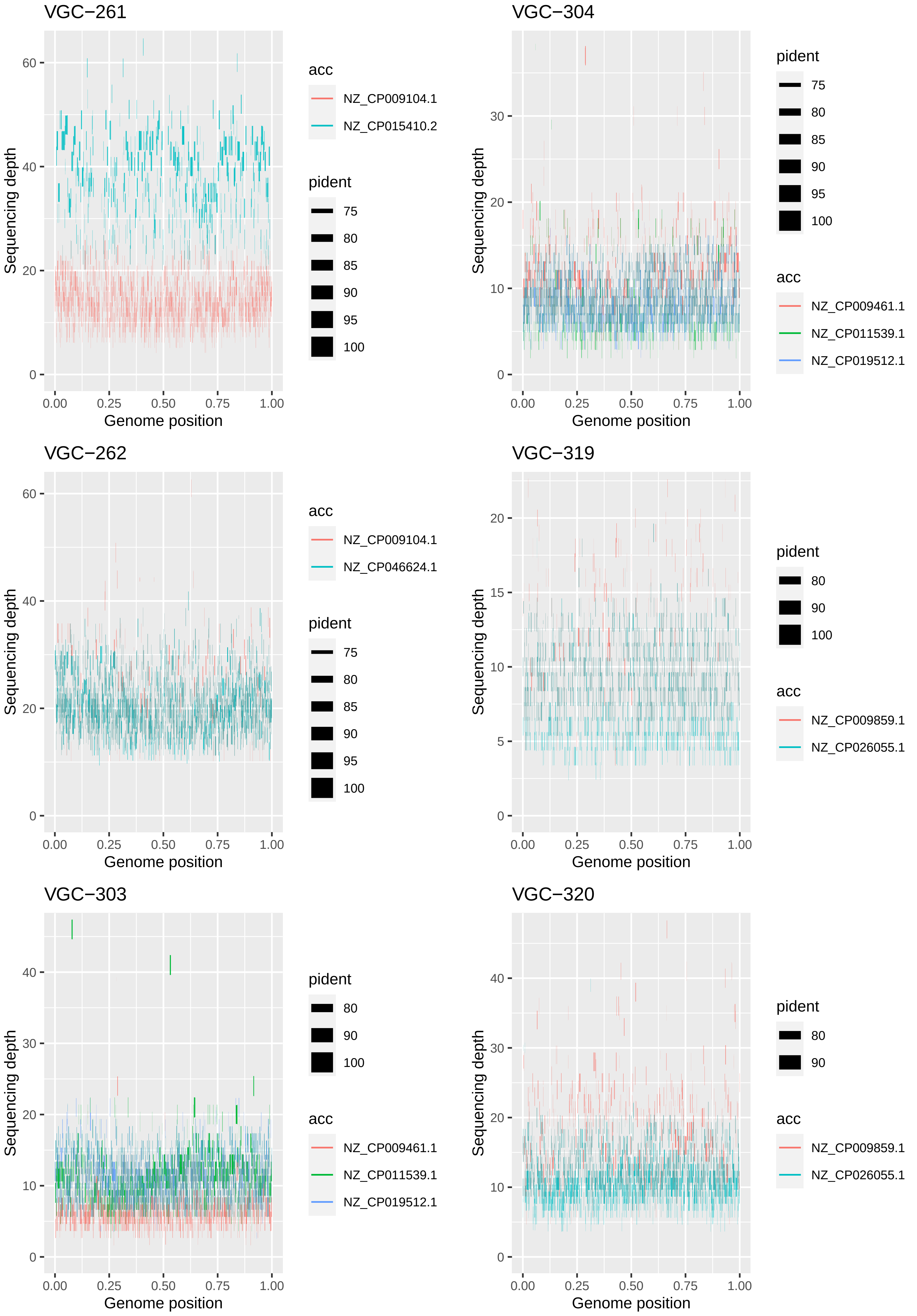
VGC−261
Sequencing depth
Genome position
0
20
40
60
0.00
0.25
0.50
0.75
1.00
acc
NZ_CP009104.1
NZ_CP015410.2
pident
75
80
85
90
95
100
VGC−304
Sequencing depth
Genome position
0
10
20
30
pident
75
80
85
90
95
100
acc
NZ_CP009461.1
NZ_CP011539.1
NZ_CP019512.1
VGC−262
Sequencing depth
Genome position
0
20
40
60
acc
NZ_CP009104.1
NZ_CP046624.1
pident
75
80
85
90
95
100
VGC−319
Sequencing depth
Genome position
0
5
10
15
20
pident
80
90
100
acc
NZ_CP009859.1
NZ_CP026055.1
VGC−303
Sequencing depth
Genome position
0
10
20
30
40
pident
80
90
100
acc
NZ_CP009461.1
NZ_CP011539.1
NZ_CP019512.1
VGC−320
Sequencing depth
Genome position
0
10
20
30
40
pident
80
90
acc
NZ_CP009859.1
NZ_CP026055.1

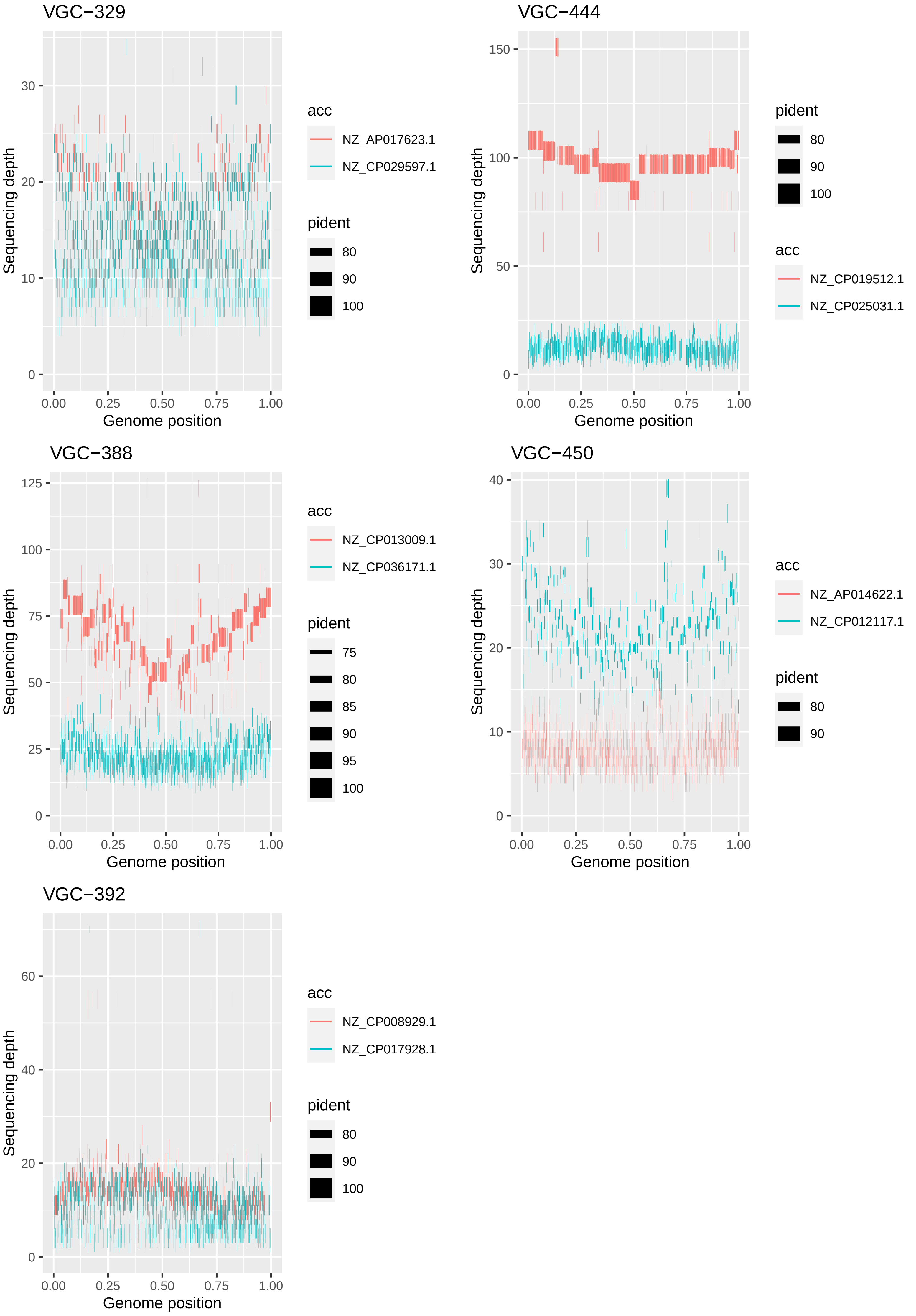

VGC−329
Sequencing depth
Genome position
0
10
20
30
0.00
0.25
0.50
0.75
1.00
acc
NZ_AP017623.1
NZ_CP029597.1
pident
80
90
100
VGC−444
Sequencing depth
Genome position
0
50
100
150
0.00
0.25
0.50
0.75
1.00
pident
80
90
100
acc
NZ_CP019512.1
NZ_CP025031.1
VGC−388
Sequencing depth
Genome position
0
25
50
75
100
125
0.00
0.25
0.50
0.75
1.00
acc
NZ_CP013009.1
NZ_CP036171.1
pident
75
80
85
90
95
100
VGC−450
Sequencing depth
Genome position
0
10
20
30
40
0.00
0.25
0.50
0.75
1.00
acc
NZ_AP014622.1
NZ_CP012117.1
pident
80
90
VGC−392
Sequencing depth
Genome position
0
20
40
60
0.00
0.25
0.50
0.75
1.00
acc
NZ_CP008929.1
NZ_CP017928.1
pident
80
90
100

Sample ID	Nanopore sequencing	MALDI-TOF MS	PacBio full-length 16S sequencing
VGC-002	Staphylococcus epidermidis; Staphylococcus caprae	Staphylococcus epidermidis	Staphylococcus epidermidis; Staphylococcus caprae
VGC-015	Escherichia coli; Klebsiella quasipneumoniae	Klebsiella pneumoniae; Escherichia coli	Klebsiella pneumoniae; Escherichia coli
VGC-040	Enterococcus faecium; Enterococcus faecalis	Enterococcus faecium; Enterococcus faecalis; Candida parapsilosis	Enterococcus faecalis; Enterococcus faecium;
VGC-049	Klebsiella pneumoniae; Enterobacter hormaechei	Klebsiella pneumoniae; Proteus mirabilis	Klebsiella pneumoniae; Proteus mirabilis; Enterobacter cloacae complex
VGC-050	Proteus mirabilis; Enterobacter hormaechei	Klebsiella pneumoniae; Proteus mirabilis; Enterobacter cloacae complex	Proteus mirabilis; Enterobacter cloacae complex
VGC-060	Escherichia coli; Klebsiella oxytoca	Klebsiella oxytoca; Escherichia coli	
VGC-063	Escherichia coli; Klebsiella oxytoca	Klebsiella oxytoca; Escherichia coli	
VGC-086	Staphylococcus epidermidis; Staphylococcus hominis	Staphylococcus not aureus	Staphylococcus hominis; Staphylococcus epidermidis
VGC-099	Escherichia coli; Enterococcus faecalis	Escherichia coli	Escherichia coli; Enterococcus faecalis
VGC-104	Enterococcus faecalis; Staphylococcus haemolyticus	Enterococcus faecalis	Enterococcus faecalis; Staphylococcus haemolyticus
VGC-105	Escherichia coli; Enterococcus faecalis	Escherichia coli; Enterococcus faecalis	
VGC-223	Staphylococcus epidermidis; Staphylococcus hominis	Staphylococcus not aureus	Staphylococcus hominis; Staphylococcus epidermidis
VGC-261	Escherichia coli; Enterococcus faecalis	Escherichia coli; Enterococcus faecalis	
VGC-262	Escherichia coli; Streptococcus sp. CNU G2	Escherichia coli; Enterococcus faecalis	Escherichia coli; Streptococcus lutetiensis
VGC-303	Klebsiella pneumoniae; Klebsiella aerogenes; Enterococcus faecalis	Klebsiella aerogenes; Enterococcus faecalis; Enterococcus avium	Klebsiella aerogenes; Klebsiella pneumoniae; Enterococcus faecalis; Enterococcus avium
VGC-304	Klebsiella pneumoniae; Klebsiella aerogenes; Enterococcus faecalis	Klebsiella aerogenes; Enterococcus faecalis; Escherichia hermannii	Klebsiella pneumoniae; Klebsiella aerogenes; Enterococcus faecalis; Enterococcus avium
VGC-319	Escherichia coli; Aeromonas caviae	Aeromonas caviae; Escherichia coli	
VGC-320	Escherichia coli; Aeromonas caviae	Aeromonas caviae; Escherichia coli	
VGC-329	Enterococcus faecalis; Klebsiella quasipneumoniae	Klebsiella pneumoniae; Enterococcus faecalis	Enterococcus faecalis; Klebsiella pneumoniae; Acinetobacter baumannii
VGC-388	Enterococcus faecium; Acinetobacter nosocomialis	Acinetobacter nosocomialis; Enterococcus faecium	
VGC-392	Klebsiella pneumoniae; Klebsiella oxytoca	Klebsiella pneumoniae; Klebsiella oxytoca	
VGC-444	Enterococcus faecalis; Staphylococcus haemolyticus	Enterococcus faecalis	Enterococcus faecalis; Staphylococcus haemolyticus
VGC-450	Pseudomonas aeruginosa; Dermabacter vaginalis	Pseudomonas aeruginosa	Dermabacter hominis; Pseudomonas aeruginosa
	The species identification is consistent between nanopore sequencing and MALDI-TOF MS		
	Samples identified as polymicrobial infections through nanopore sequencing		
	The species identification is consistent between nanopore and pacbio sequencing		

VGC-002

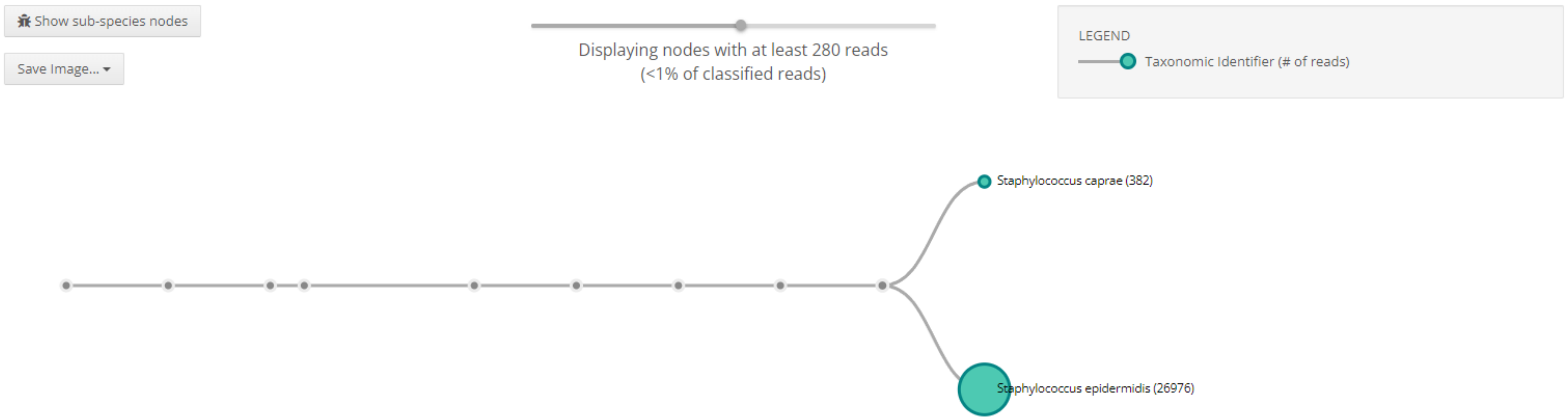

VGC-015

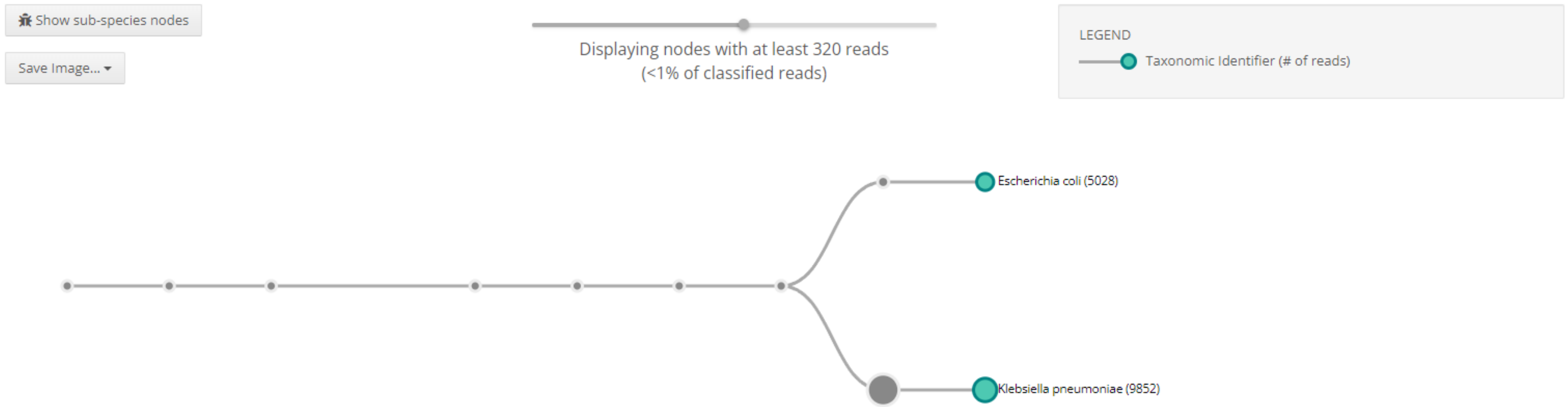

VGC-040

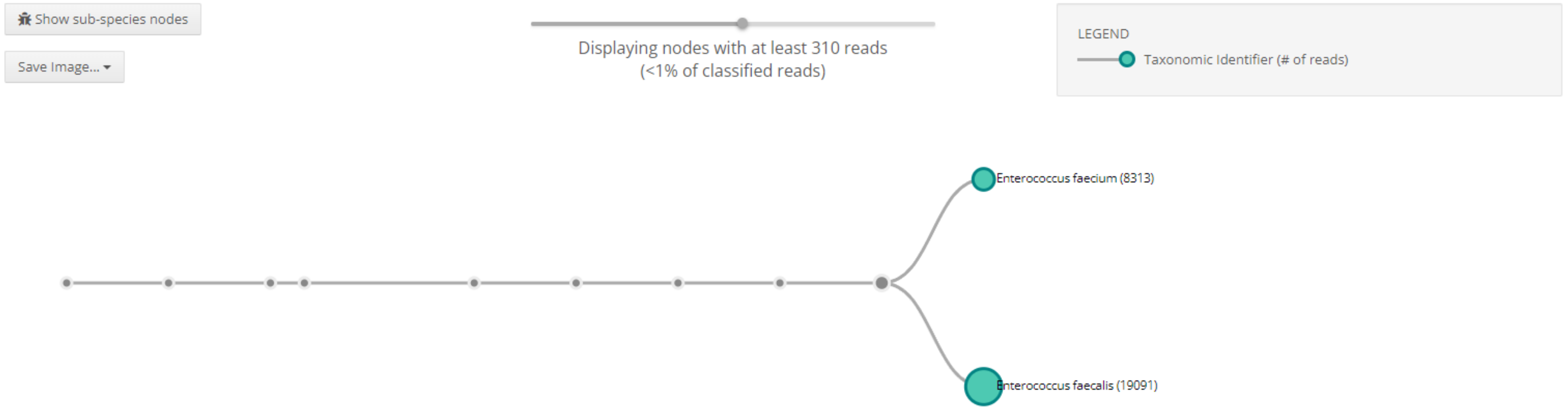

VGC-049

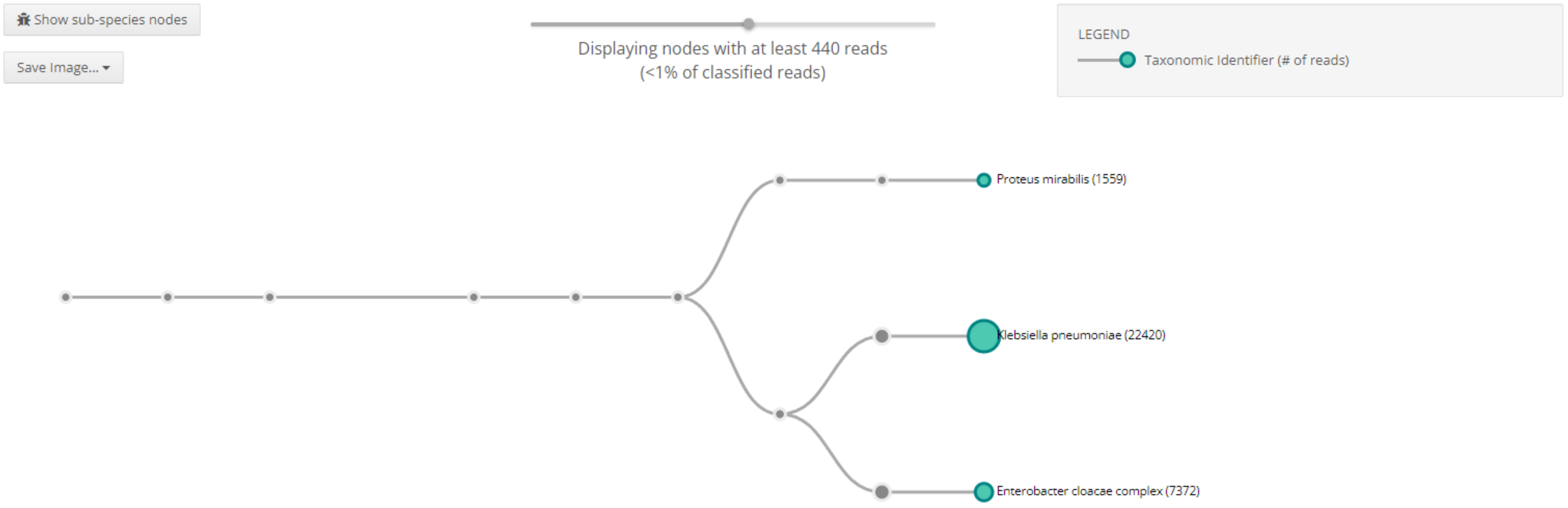

VGC-050

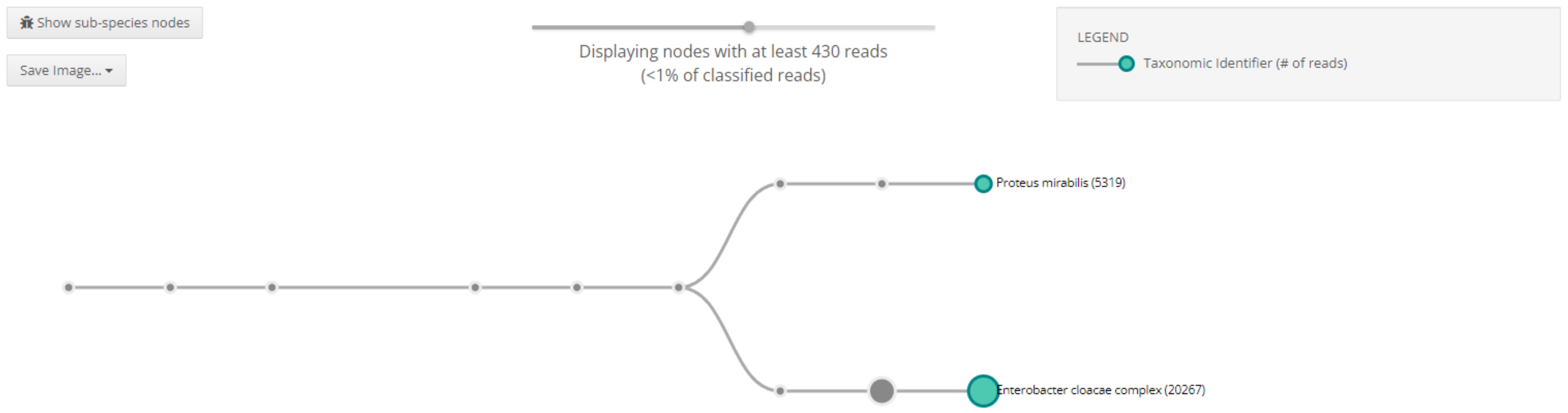

VGC-086

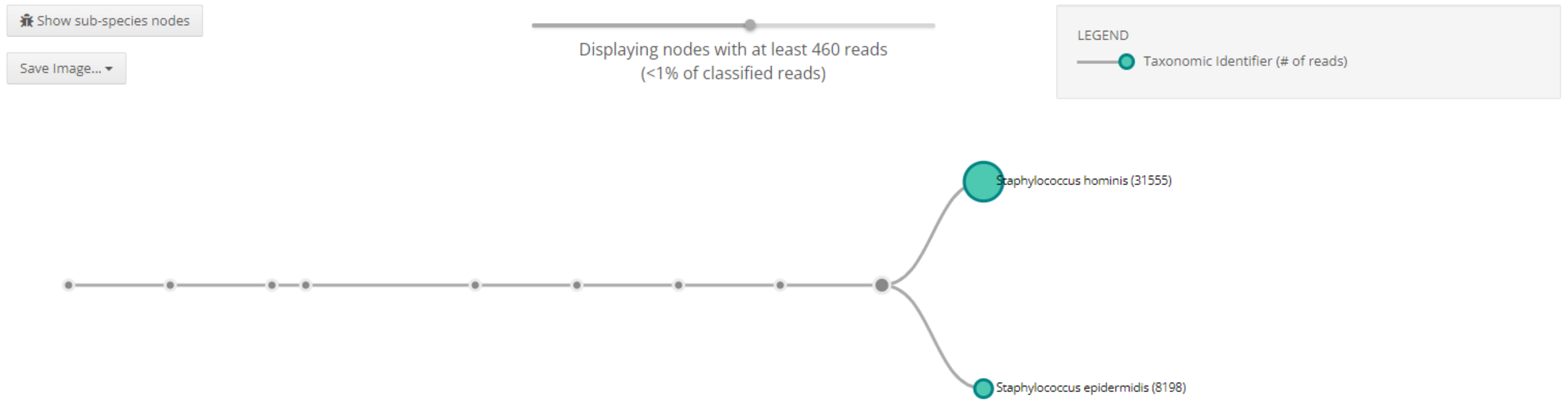

VGC-099

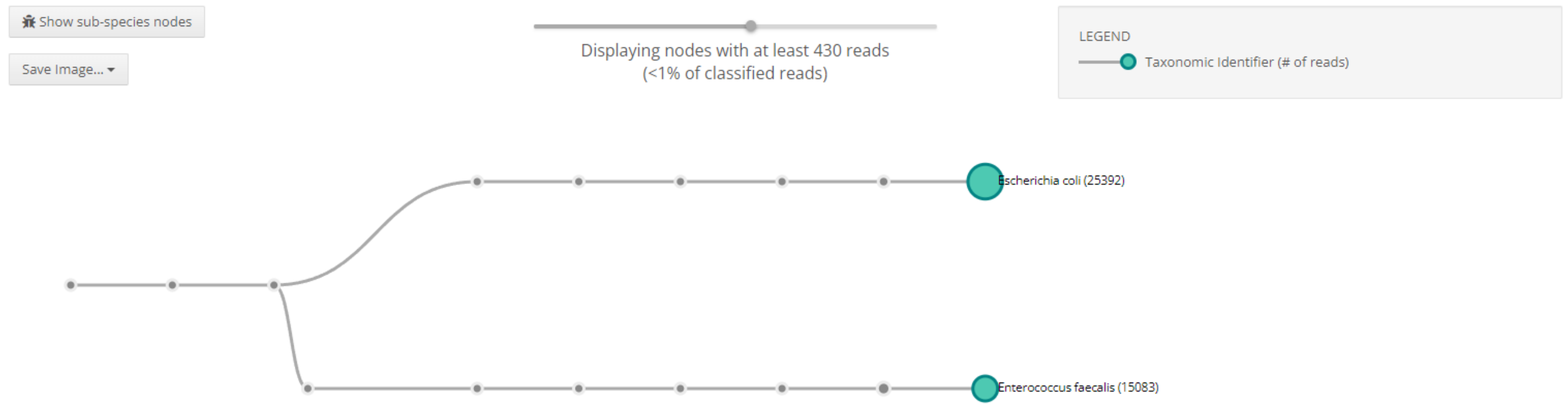

VGC-104

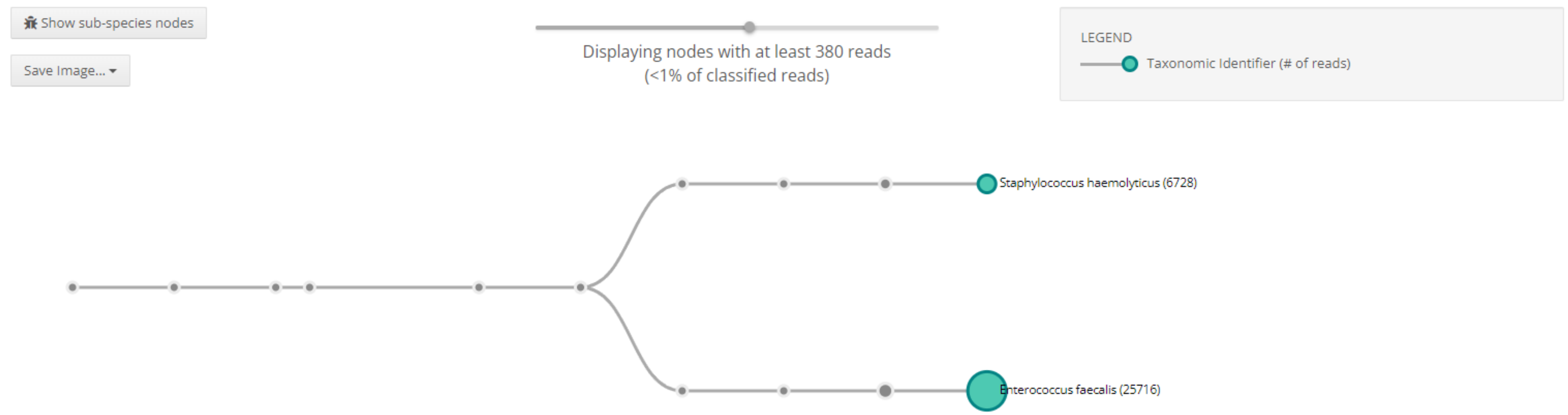

VGC-223

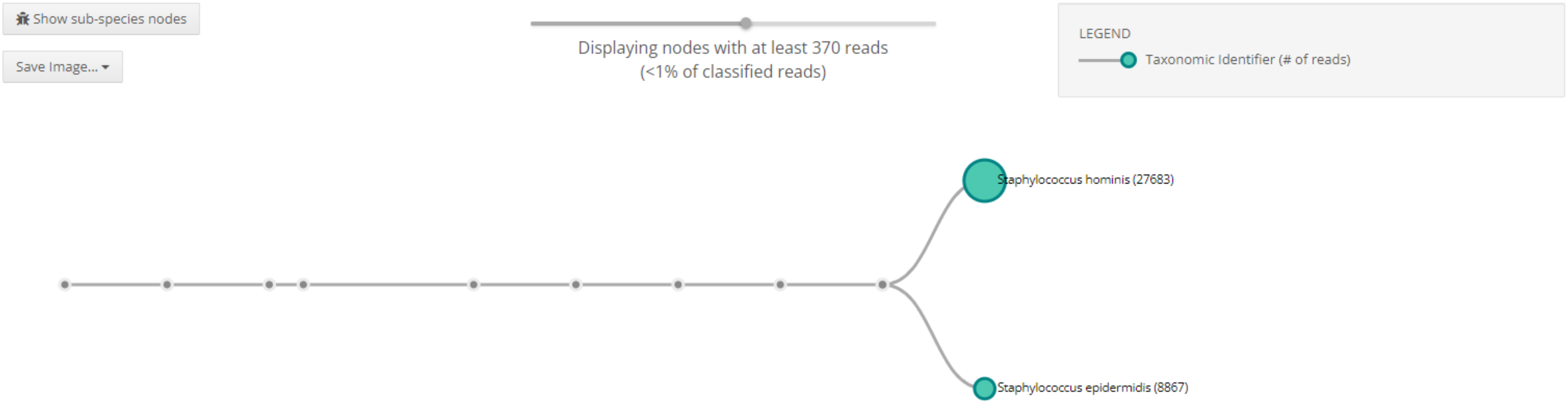

VGC-262

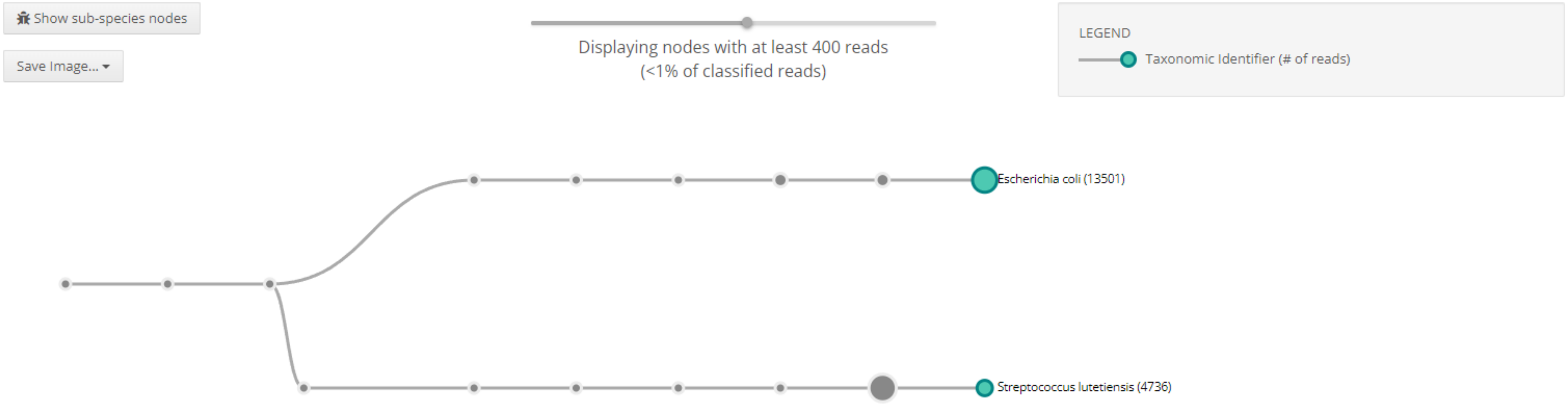

VGC-303

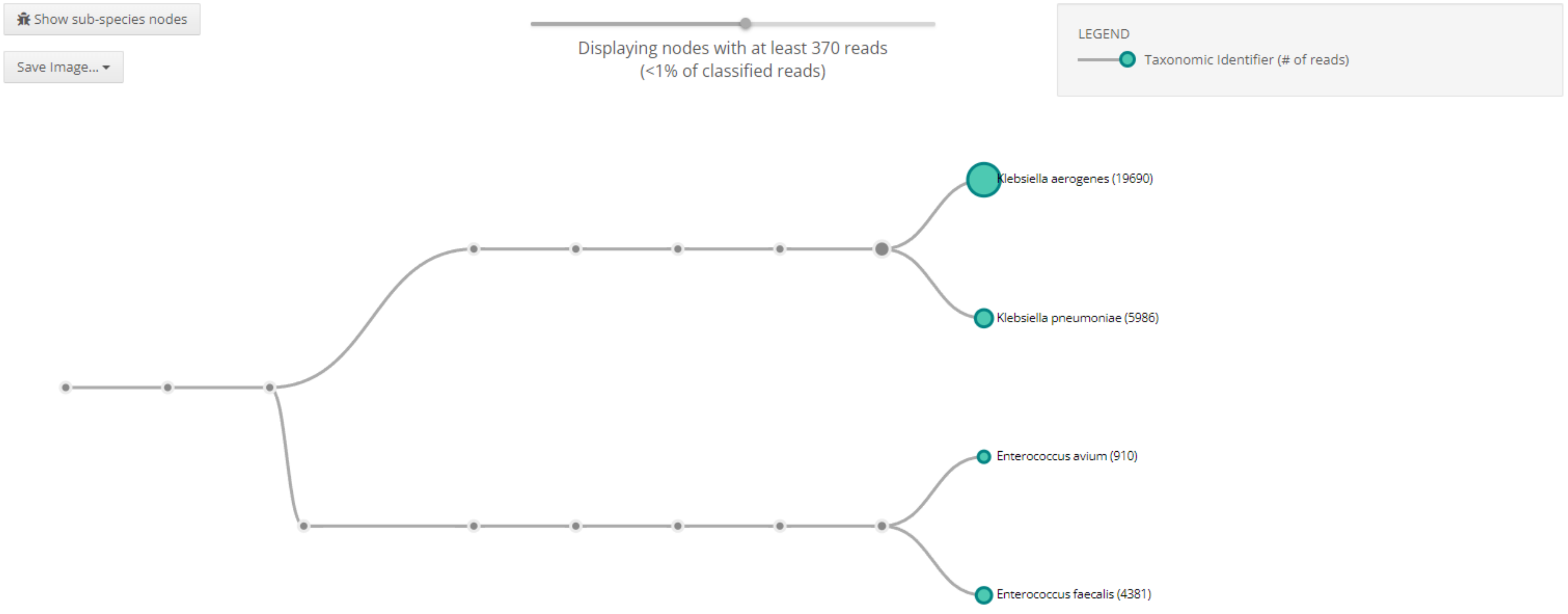
Show sub-species nodes
Save Image...
Displaying nodes with at least 370 reads
(<1% of classified reads)
LEGEND
Taxonomic Identifier (# of reads)
Klebsiella aerogenes (19690)
Klebsiella pneumoniae (5986)
Enterococcus avium (910)
Enterococcus faecalis (4381)

VGC-304

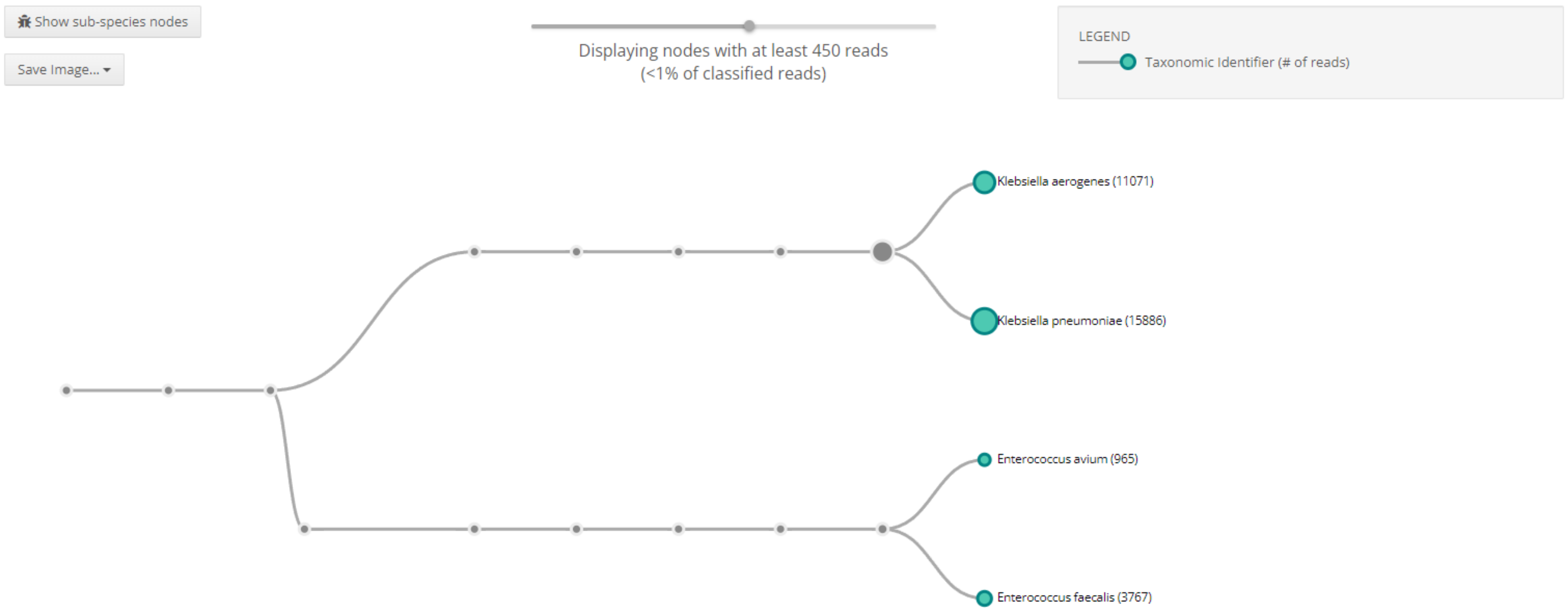

Show sub-species nodes
Save Image...
Displaying nodes with at least 450 reads
(<1% of classified reads)
LEGEND
Taxonomic Identifier (# of reads)
Klebsiella aerogenes (11071)
Klebsiella pneumoniae (15886)
Enterococcus avium (965)
Enterococcus faecalis (3767)

VGC-329

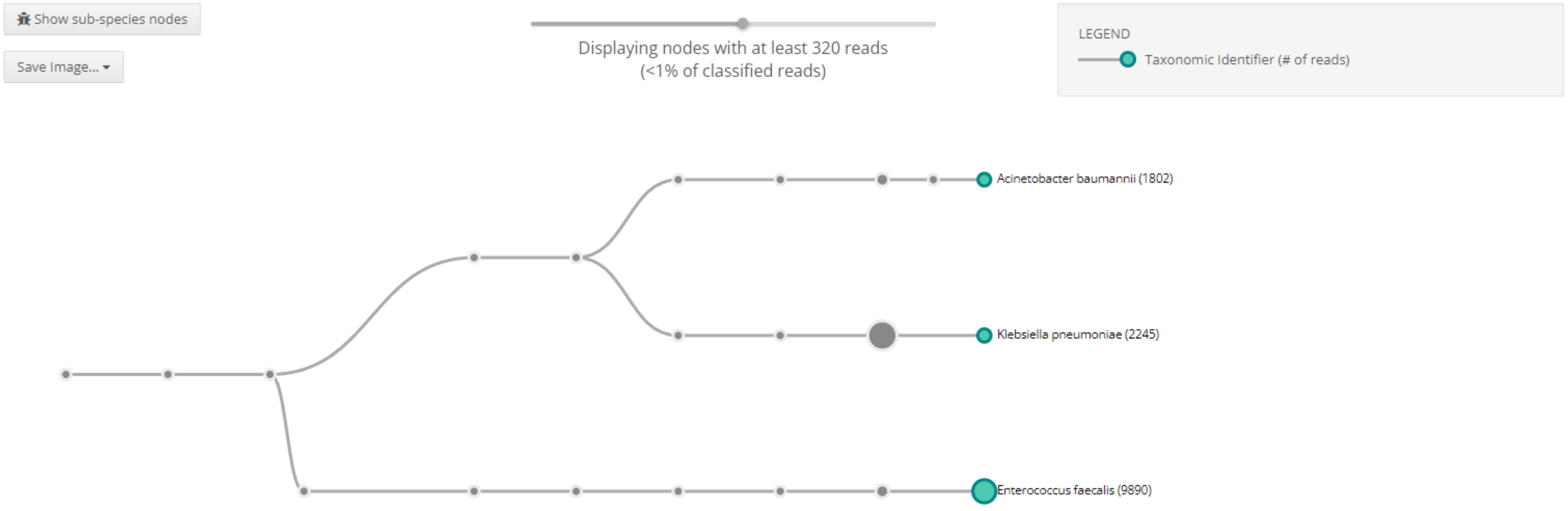

VGC-444

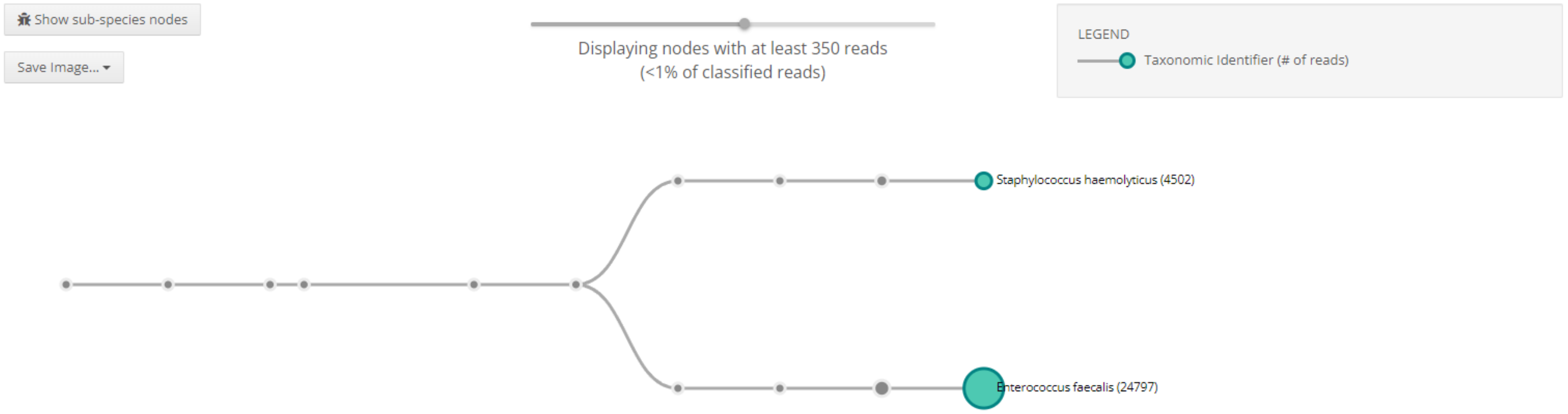

VGC-450

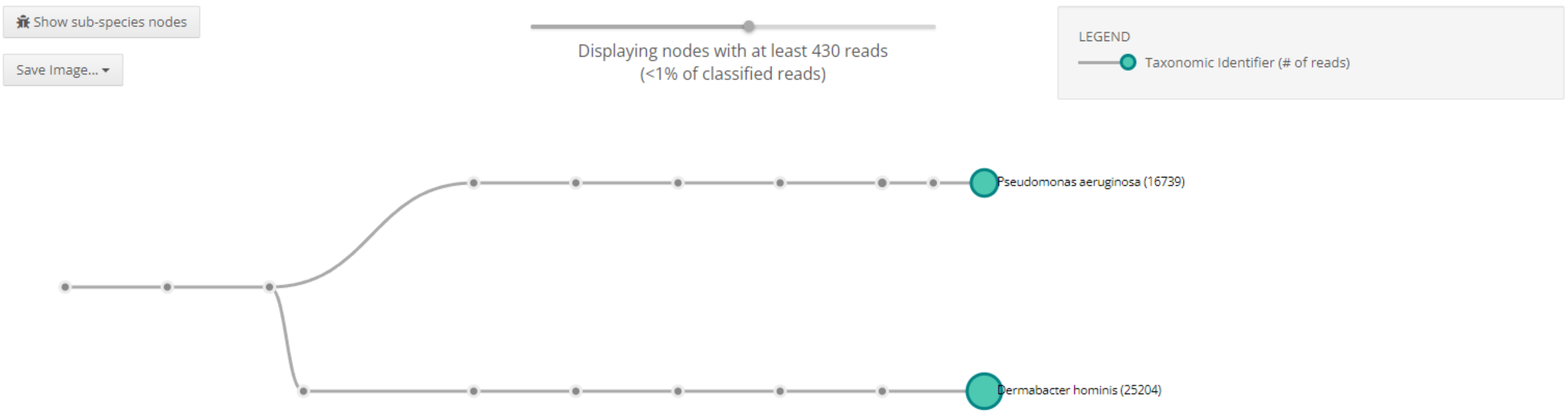

Show sub-species nodes
Save Image...
Displaying nodes with at least 430 reads
(<1% of classified reads)
LEGEND
Taxonomic Identifier (# of reads)
Pseudomonas aeruginosa (16739)
Dermabacter hominis (25204)

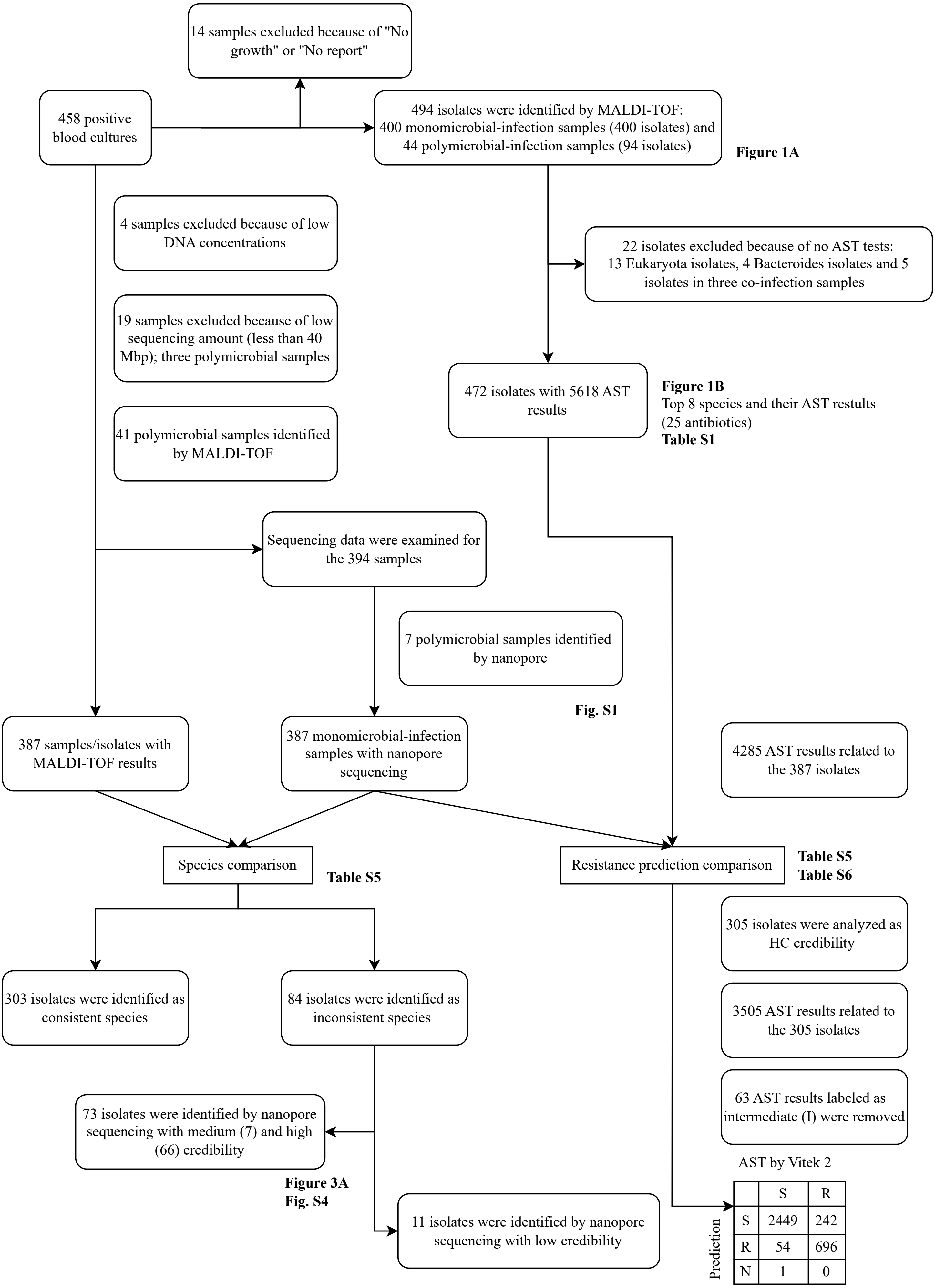
14 samples excluded because of "No growth" or "No report"
458 positive blood cultures
494 isolates were identified by MALDI-TOF: 400 monomicrobial-infection samples (400 isolates) and 44 polymicrobial-infection samples (94 isolates)
Figure 1A
4 samples excluded because of low DNA concentrations
22 isolates excluded because of no AST tests: 13 Eukaryota isolates, 4 Bacteroides isolates and 5 isolates in three co-infection samples
19 samples excluded because of low sequencing amount (less than 40 Mbp); three polymicrobial samples
472 isolates with 5618 AST results
Figure 1B
Top 8 species and their AST restults (25 antibiotics)
Table S1
41 polymicrobial samples identified by MALDI-TOF
Sequencing data were examined for the 394 samples
7 polymicrobial samples identified by nanopore
Fig. S1
387 samples/isolates with MALDI-TOF results
387 monomicrobial-infection samples with nanopore sequencing
4285 AST results related to the 387 isolates
Species comparison
Table S5
Resistance prediction comparison
Table S5
Table S6
305 isolates were analyzed as HC credibility
303 isolates were identified as consistent species
84 isolates were identified as inconsistent species
3505 AST results related to the 305 isolates
73 isolates were identified by nanopore sequencing with medium (7) and high (66) credibility
63 AST results labeled as intermediate (I) were removed
AST by Vitek 2
Figure 3A
Fig. S4
11 isolates were identified by nanopore sequencing with low credibility
Prediction
S
R
S 2449 242
R 54 696
N 1 0

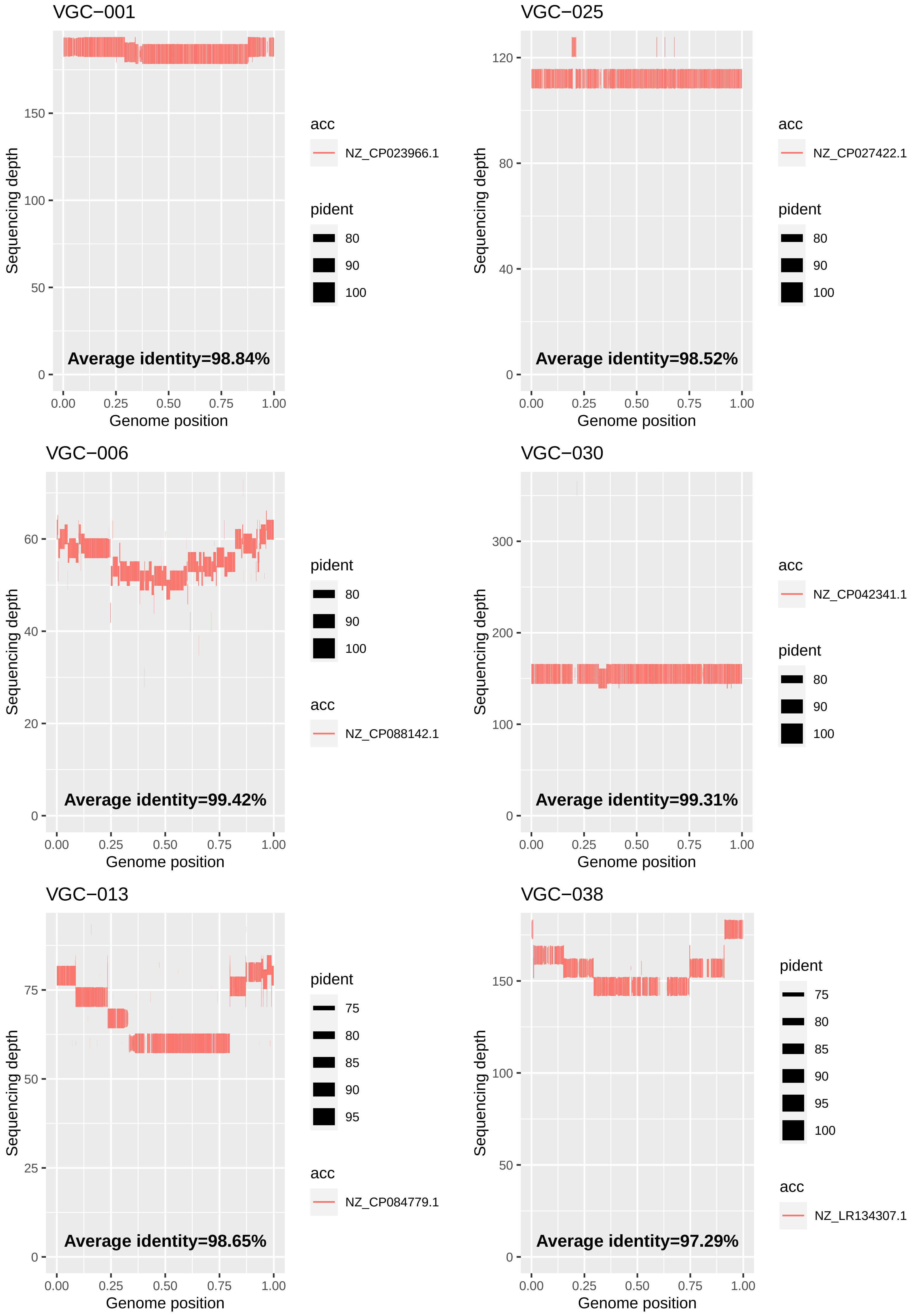
VGC−001
Sequencing depth
Genome position
Average identity=98.84%
acc
NZ_CP023966.1
pident
80
90
100
VGC−025
Average identity=98.52%
NZ_CP027422.1
VGC−006
Average identity=99.42%
NZ_CP088142.1
VGC−030
Average identity=99.31%
NZ_CP042341.1
VGC−013
Average identity=98.65%
75
85
95
NZ_CP084779.1
VGC−038
Average identity=97.29%
NZ_LR134307.1
0.00
0.25
0.50
0.75
1.00

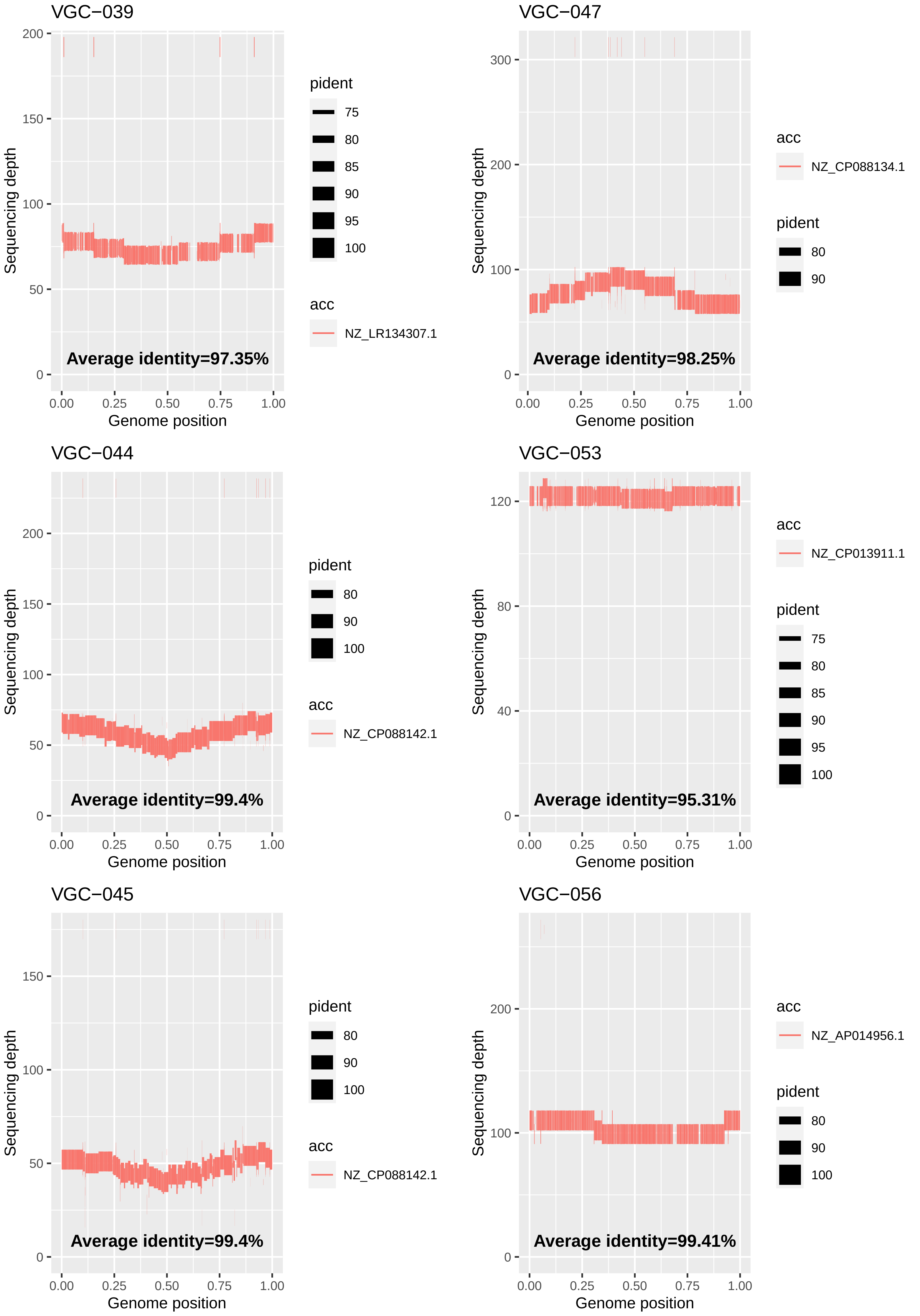

VGC−039
Sequencing depth
Genome position
Average identity=97.35%
pident
75
80
85
90
95
100
acc
NZ_LR134307.1
VGC−047
Sequencing depth
Genome position
Average identity=98.25%
acc
NZ_CP088134.1
pident
80
90
VGC−044
Sequencing depth
Genome position
Average identity=99.4%
pident
80
90
100
acc
NZ_CP088142.1
VGC−053
Sequencing depth
Genome position
Average identity=95.31%
acc
NZ_CP013911.1
pident
75
80
85
90
95
100
VGC−045
Sequencing depth
Genome position
Average identity=99.4%
pident
80
90
100
acc
NZ_CP088142.1
VGC−056
Sequencing depth
Genome position
Average identity=99.41%
acc
NZ_AP014956.1
pident
80
90
100

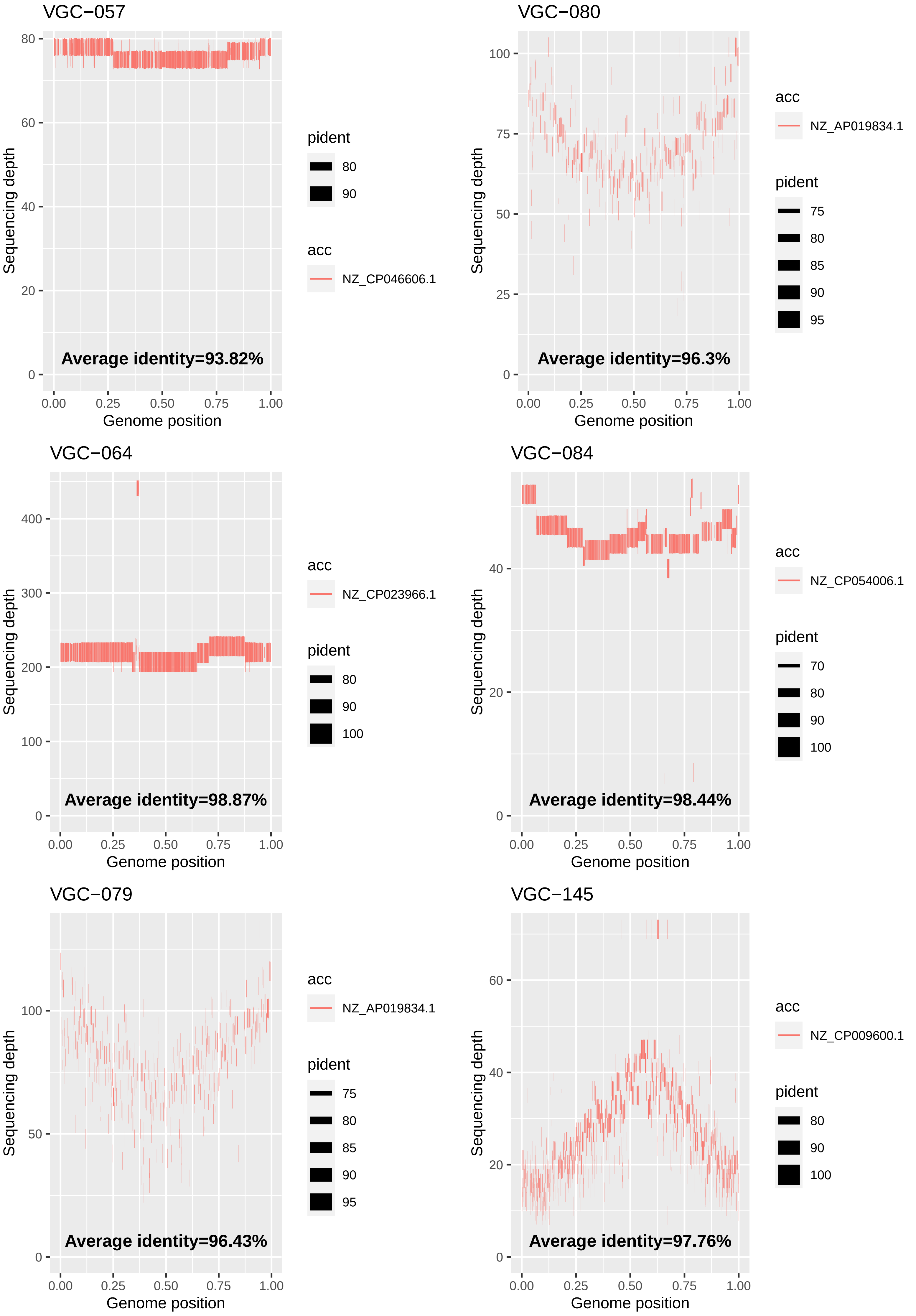
VGC−057
Sequencing depth
Genome position
Average identity=93.82%
pident
80
90
acc
NZ_CP046606.1
VGC−080
Sequencing depth
Genome position
Average identity=96.3%
acc
NZ_AP019834.1
pident
75
80
85
90
95
VGC−064
Sequencing depth
Genome position
Average identity=98.87%
acc
NZ_CP023966.1
pident
80
90
100
VGC−084
Sequencing depth
Genome position
Average identity=98.44%
acc
NZ_CP054006.1
pident
70
80
90
100
VGC−079
Sequencing depth
Genome position
Average identity=96.43%
acc
NZ_AP019834.1
pident
75
80
85
90
95
VGC−145
Sequencing depth
Genome position
Average identity=97.76%
acc
NZ_CP009600.1
pident
80
90
100

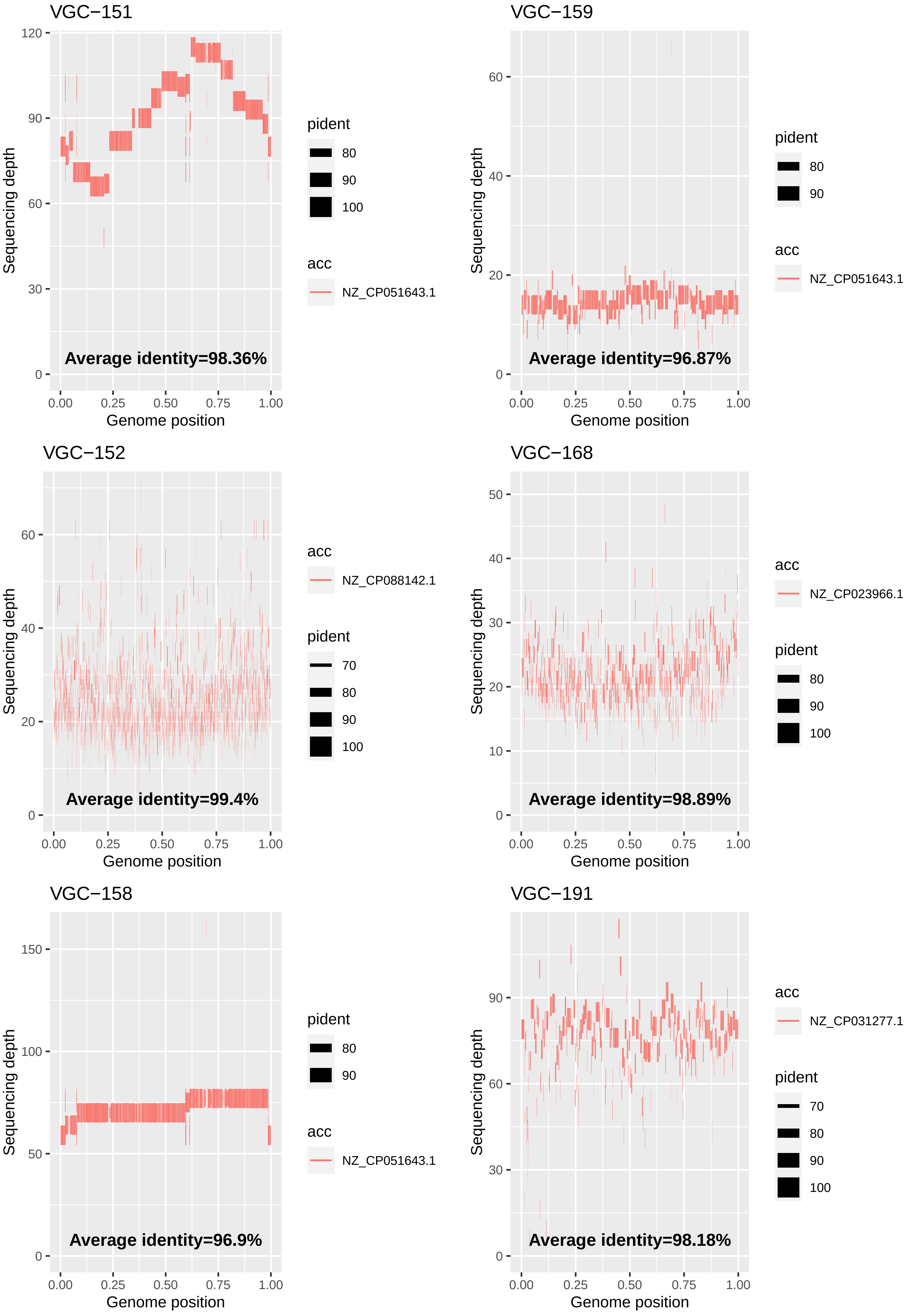
VGC−151
Sequencing depth
Genome position
Average identity=98.36%
pident
acc
NZ_CP051643.1
VGC−159
Average identity=96.87%
VGC−152
Average identity=99.4%
NZ_CP088142.1
VGC−168
Average identity=98.89%
NZ_CP023966.1
VGC−158
Average identity=96.9%
VGC−191
Average identity=98.18%
NZ_CP031277.1

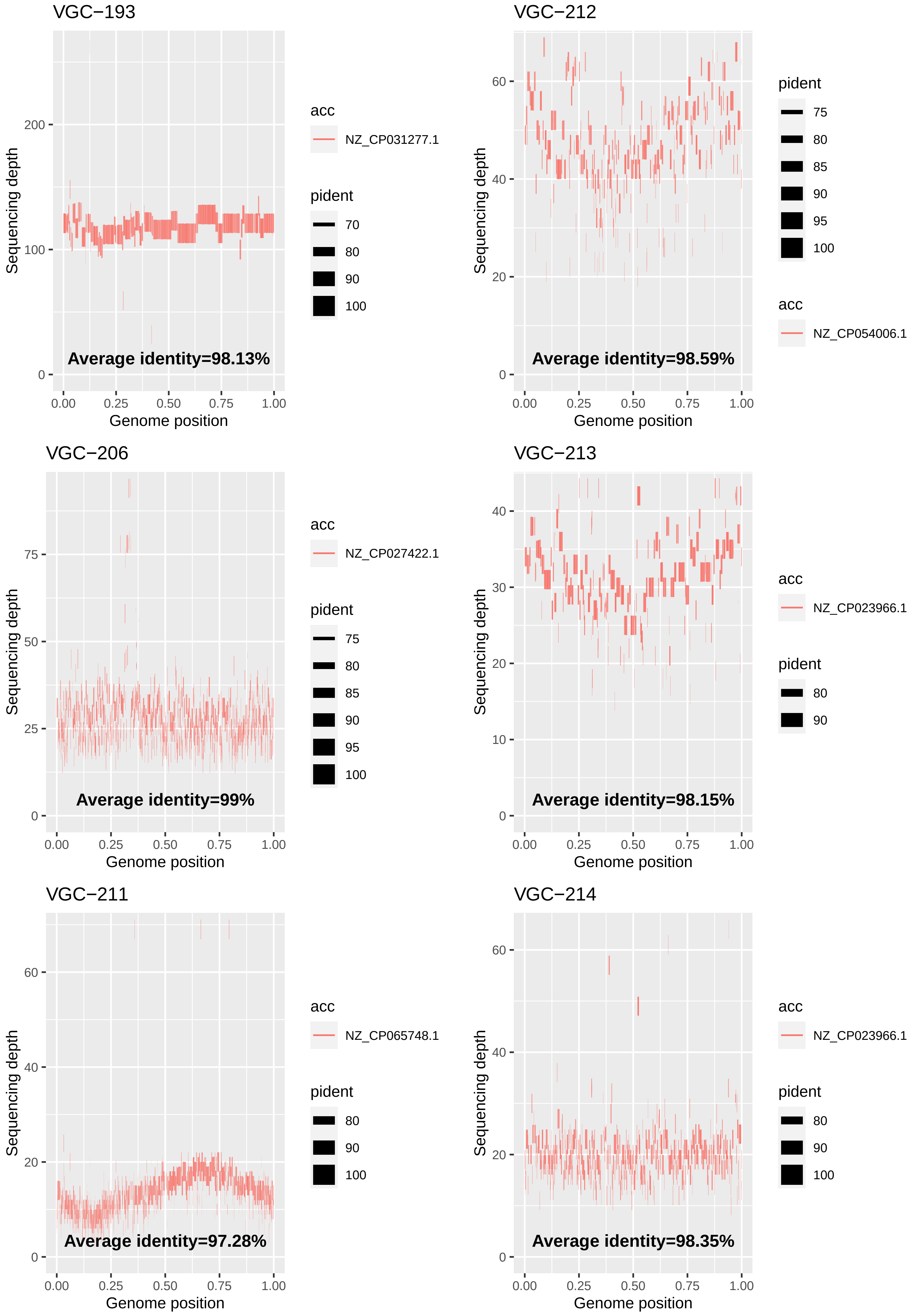

VGC−193
Sequencing depth
Genome position
0
100
200
0.00
0.25
0.50
0.75
1.00
Average identity=98.13%
acc
NZ_CP031277.1
pident
70
80
90
100
VGC−212
Sequencing depth
Genome position
0
20
40
60
0.00
0.25
0.50
0.75
1.00
Average identity=98.59%
pident
75
80
85
90
95
100
acc
NZ_CP054006.1
VGC−206
Sequencing depth
Genome position
0
25
50
75
0.00
0.25
0.50
0.75
1.00
Average identity=99%
acc
NZ_CP027422.1
pident
75
80
85
90
95
100
VGC−213
Sequencing depth
Genome position
0
10
20
30
40
0.00
0.25
0.50
0.75
1.00
Average identity=98.15%
acc
NZ_CP023966.1
pident
80
90
VGC−211
Sequencing depth
Genome position
0
20
40
60
0.00
0.25
0.50
0.75
1.00
Average identity=97.28%
acc
NZ_CP065748.1
pident
80
90
100
VGC−214
Sequencing depth
Genome position
0
20
40
60
0.00
0.25
0.50
0.75
1.00
Average identity=98.35%
acc
NZ_CP023966.1
pident
80
90
100

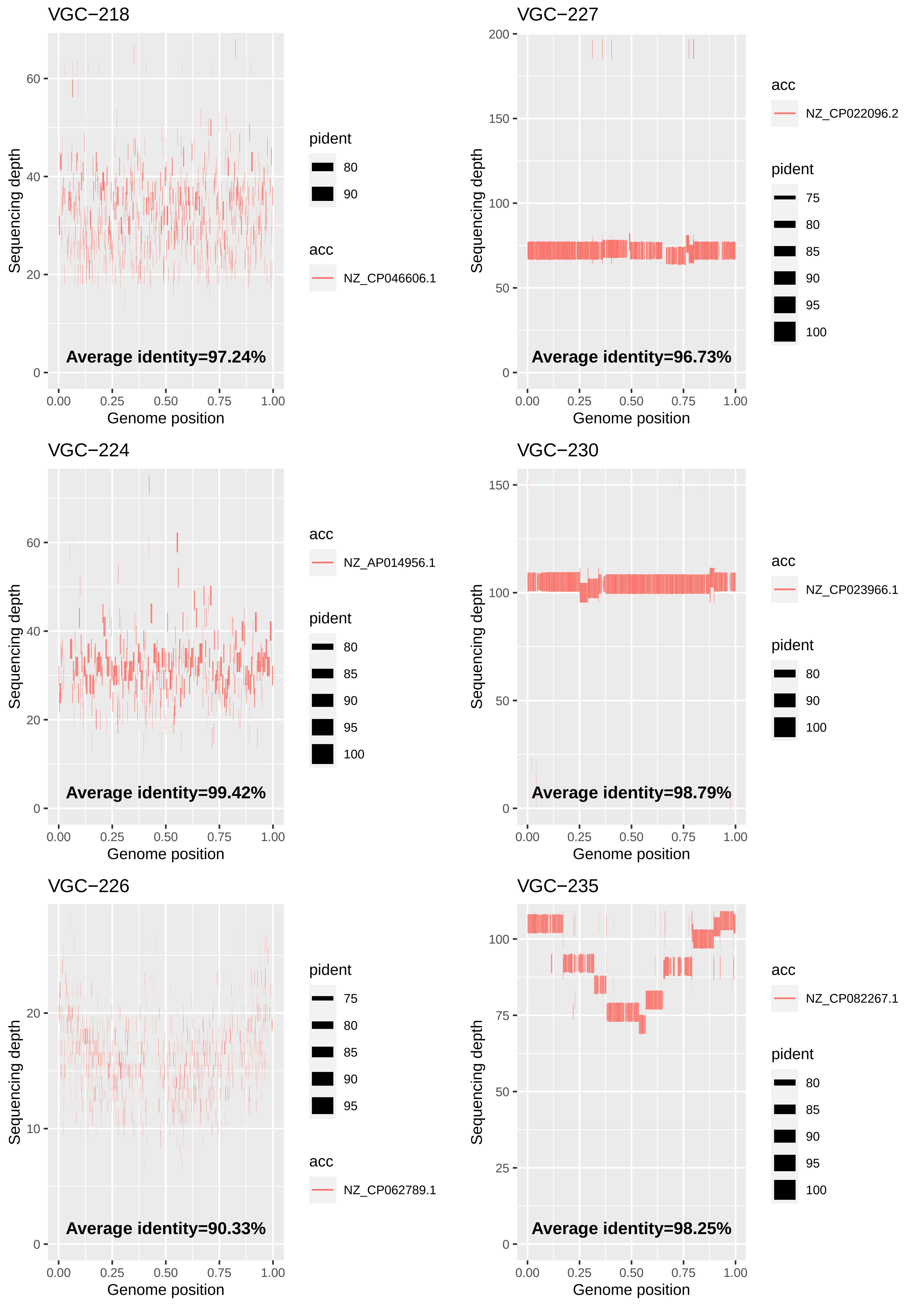

VGC−218
Sequencing depth
Genome position
Average identity=97.24%
pident
80
90
acc
NZ_CP046606.1
VGC−227
Average identity=96.73%
acc
NZ_CP022096.2
pident
75
80
85
90
95
100
VGC−224
Average identity=99.42%
acc
NZ_AP014956.1
pident
80
85
90
95
100
VGC−230
Average identity=98.79%
acc
NZ_CP023966.1
pident
80
90
100
VGC−226
Average identity=90.33%
pident
75
80
85
90
95
acc
NZ_CP062789.1
VGC−235
Average identity=98.25%
acc
NZ_CP082267.1
pident
80
85
90
95
100

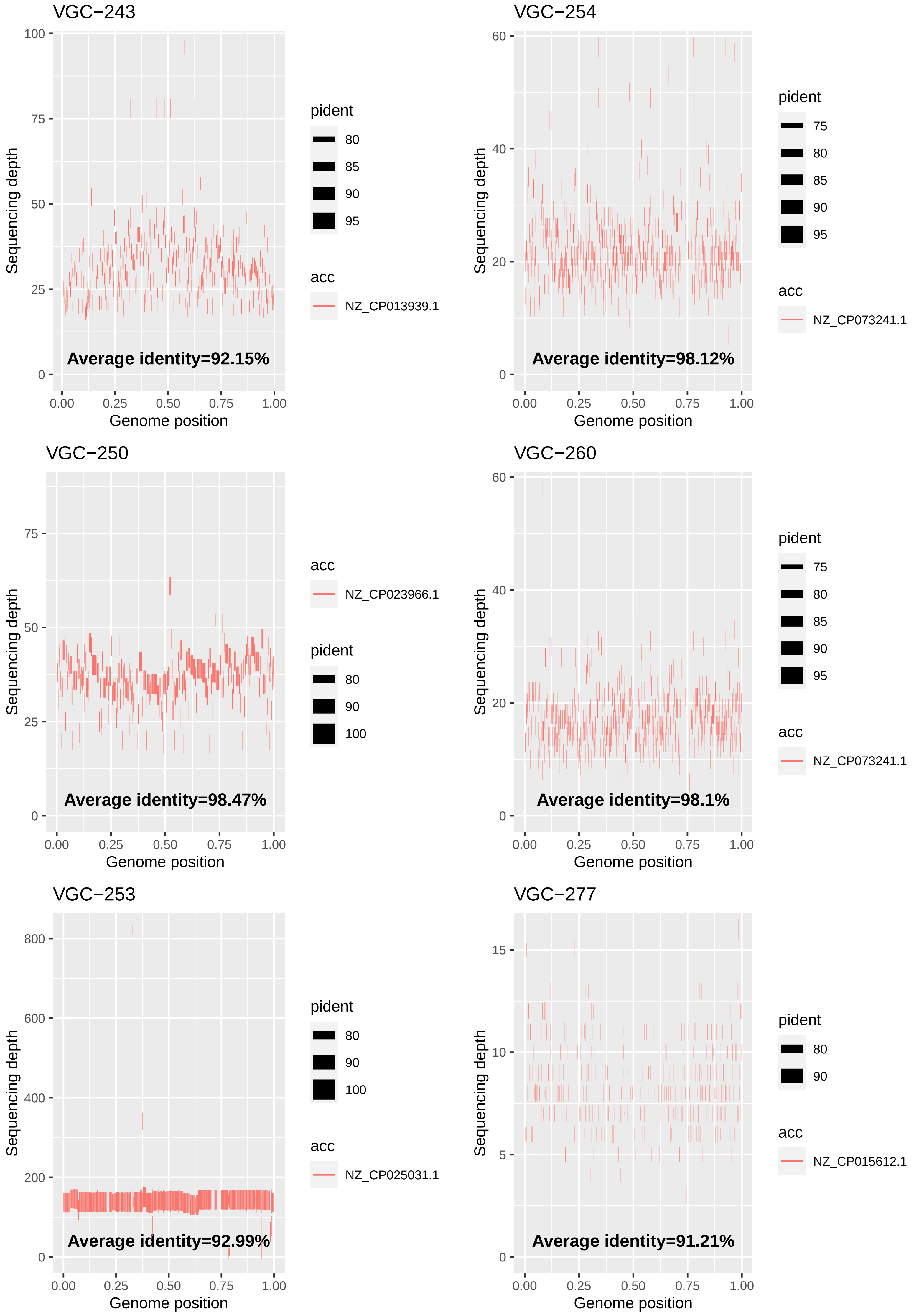
VGC−243
Sequencing depth
Genome position
pident
80
85
90
95
acc
NZ_CP013939.1
Average identity=92.15%
VGC−254
pident
75
80
85
90
95
acc
NZ_CP073241.1
Average identity=98.12%
VGC−250
acc
NZ_CP023966.1
pident
80
90
100
Average identity=98.47%
VGC−260
pident
75
80
85
90
95
acc
NZ_CP073241.1
Average identity=98.1%
VGC−253
pident
80
90
100
acc
NZ_CP025031.1
Average identity=92.99%
VGC−277
pident
80
90
acc
NZ_CP015612.1
Average identity=91.21%

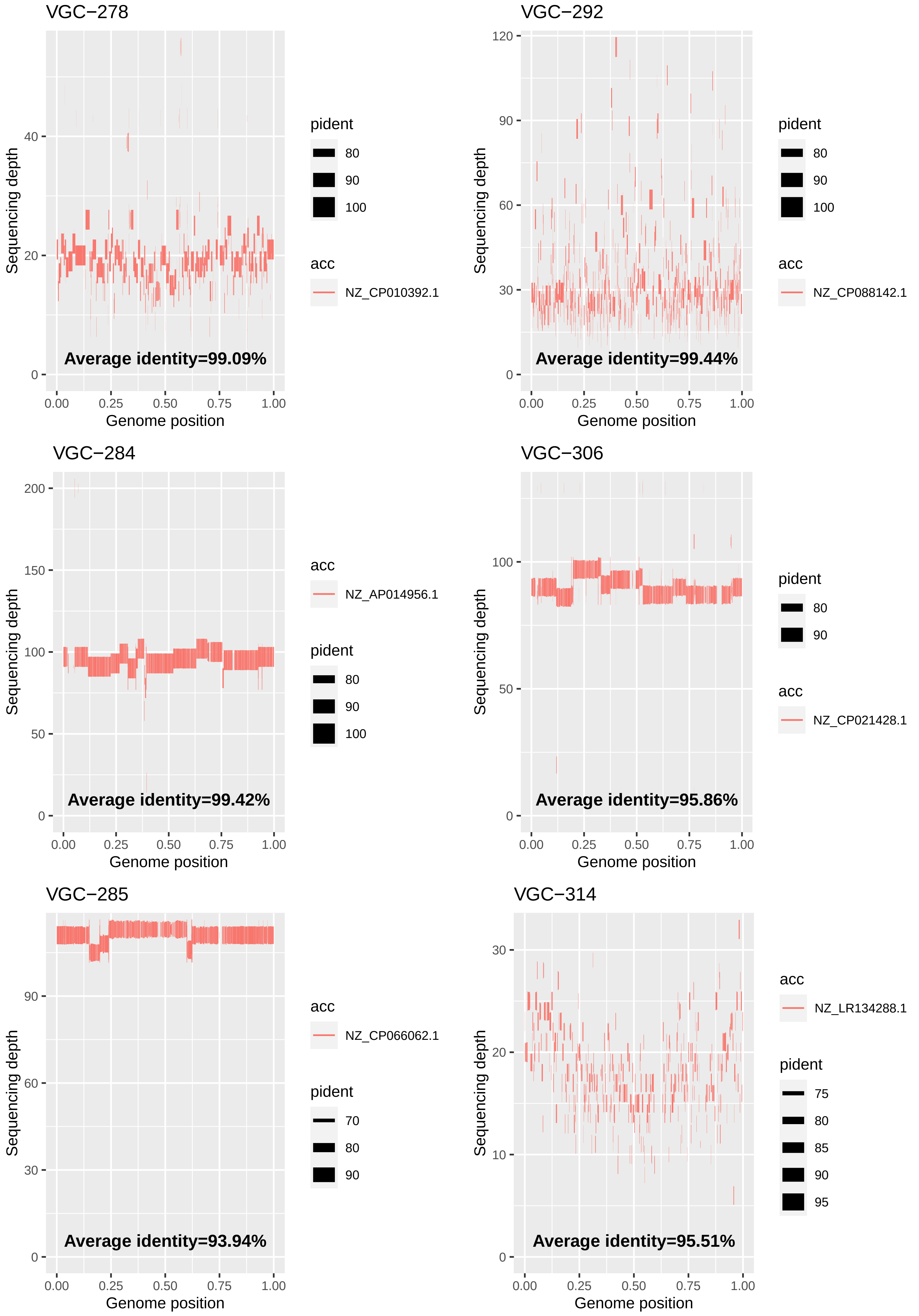

VGC−278
Sequencing depth
Genome position
0.00 0.25 0.50 0.75 1.00
0 20 40
pident
80 90 100
acc
NZ_CP010392.1
Average identity=99.09%
VGC−292
Sequencing depth
Genome position
0.00 0.25 0.50 0.75 1.00
0 30 60 90 120
pident
80 90 100
acc
NZ_CP088142.1
Average identity=99.44%
VGC−284
Sequencing depth
Genome position
0.00 0.25 0.50 0.75 1.00
0 50 100 150 200
acc
NZ_AP014956.1
pident
80 90 100
Average identity=99.42%
VGC−306
Sequencing depth
Genome position
0.00 0.25 0.50 0.75 1.00
0 50 100
pident
80 90
acc
NZ_CP021428.1
Average identity=95.86%
VGC−285
Sequencing depth
Genome position
0.00 0.25 0.50 0.75 1.00
0 30 60 90
acc
NZ_CP066062.1
pident
70 80 90
Average identity=93.94%
VGC−314
Sequencing depth
Genome position
0.00 0.25 0.50 0.75 1.00
0 10 20 30
acc
NZ_LR134288.1
pident
75 80 85 90 95
Average identity=95.51%

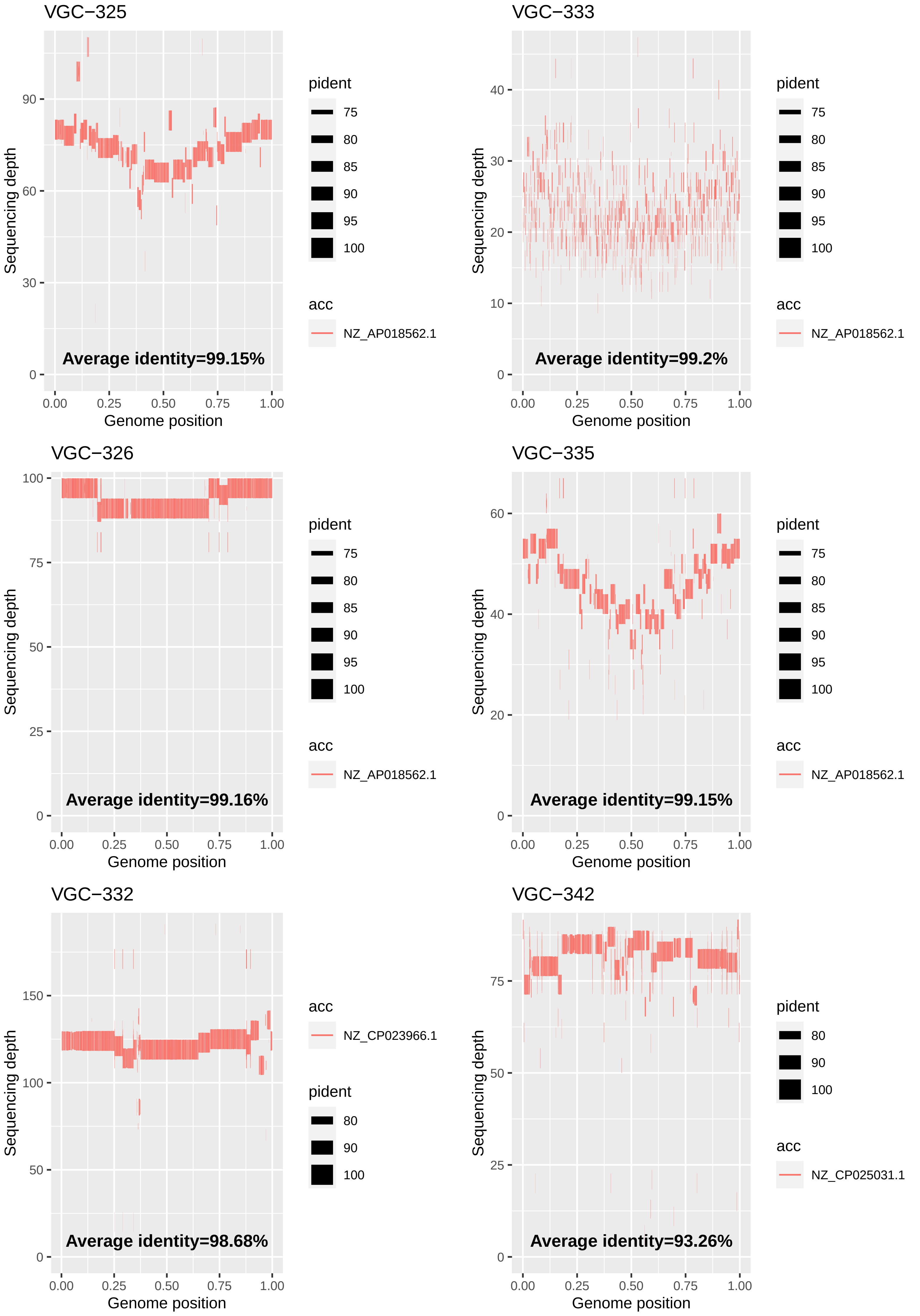
VGC−325
Average identity=99.15%
VGC−333
Average identity=99.2%
VGC−326
Average identity=99.16%
VGC−335
Average identity=99.15%
VGC−332
Average identity=98.68%
VGC−342
Average identity=93.26%
Sequencing depth
Genome position
pident
75
80
85
90
95
100
acc
NZ_AP018562.1
NZ_CP023966.1
NZ_CP025031.1

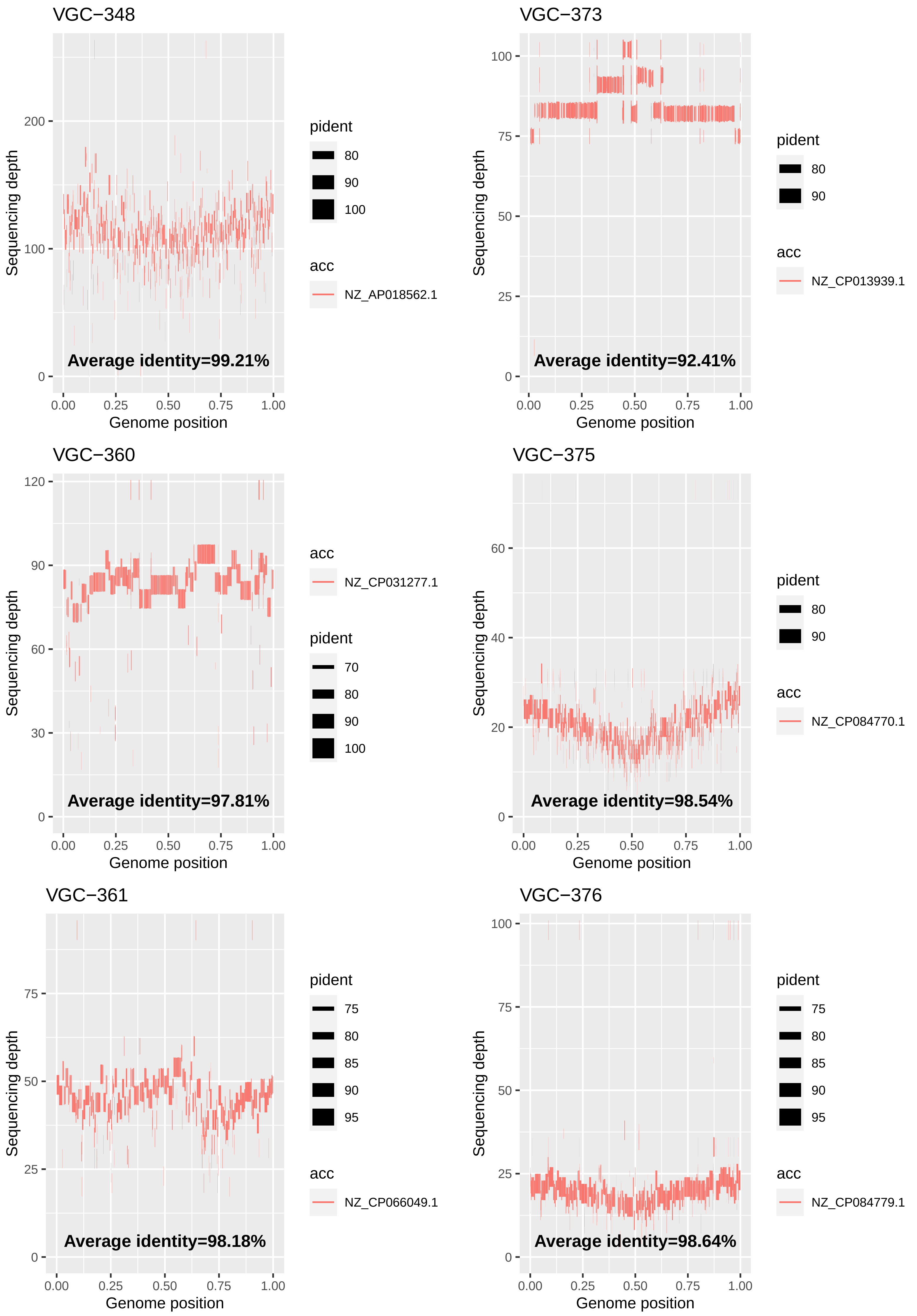

VGC−348
Sequencing depth
0
100
200
Average identity=99.21%
0.00
0.25
0.50
0.75
1.00
Genome position
pident
80
90
100
acc
NZ_AP018562.1
VGC−373
Sequencing depth
0
25
50
75
100
Average identity=92.41%
0.00
0.25
0.50
0.75
1.00
Genome position
pident
80
90
acc
NZ_CP013939.1
VGC−360
Sequencing depth
0
30
60
90
120
Average identity=97.81%
0.00
0.25
0.50
0.75
1.00
Genome position
acc
NZ_CP031277.1
pident
70
80
90
100
VGC−375
Sequencing depth
0
20
40
60
Average identity=98.54%
0.00
0.25
0.50
0.75
1.00
Genome position
pident
80
90
acc
NZ_CP084770.1
VGC−361
Sequencing depth
0
25
50
75
Average identity=98.18%
0.00
0.25
0.50
0.75
1.00
Genome position
pident
75
80
85
90
95
acc
NZ_CP066049.1
VGC−376
Sequencing depth
0
25
50
75
100
Average identity=98.64%
0.00
0.25
0.50
0.75
1.00
Genome position
pident
75
80
85
90
95
acc
NZ_CP084779.1

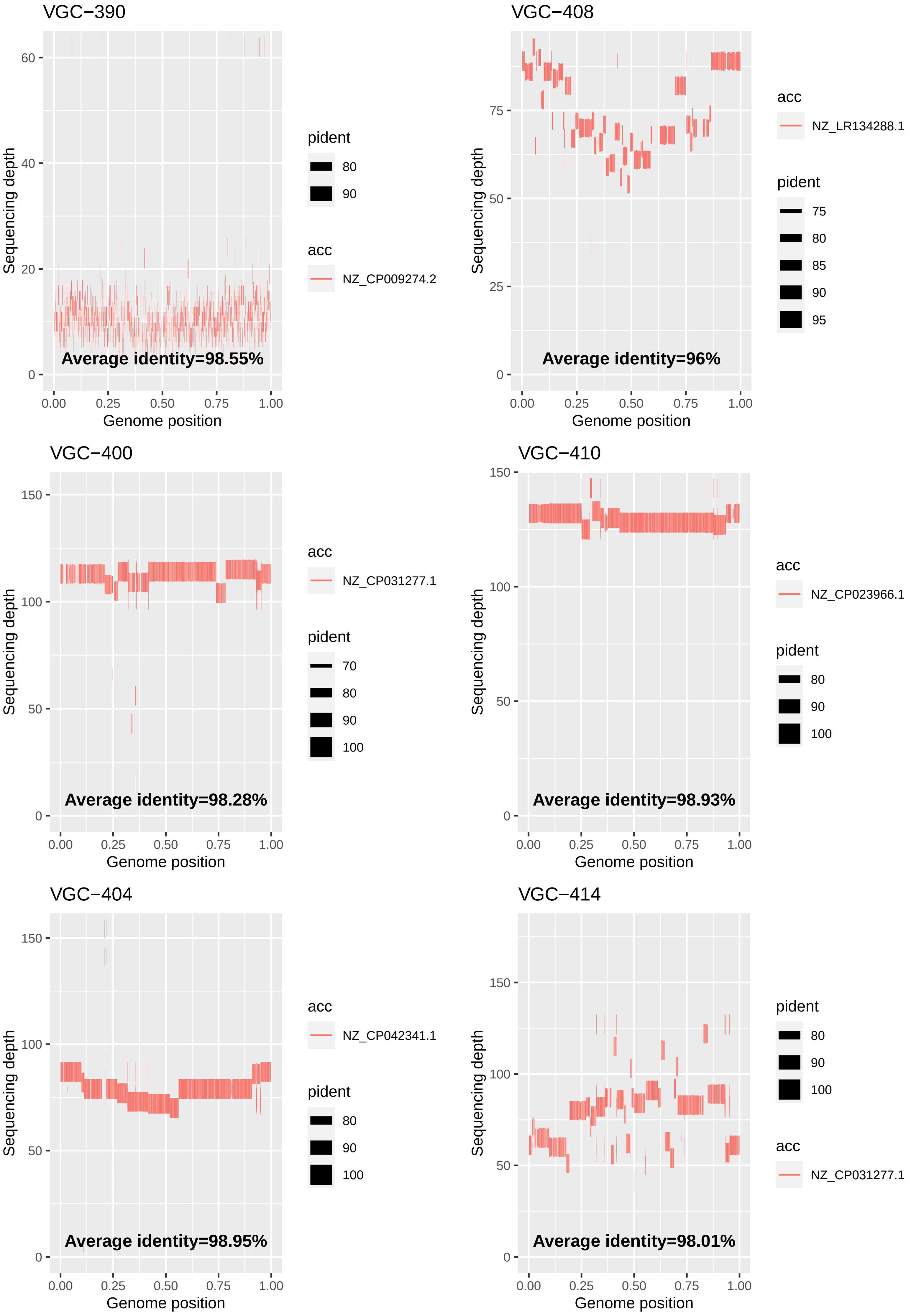
VGC−390
Sequencing depth
Genome position
pident
80
90
acc
NZ_CP009274.2
Average identity=98.55%
VGC−408
acc
NZ_LR134288.1
pident
75
80
85
90
95
Average identity=96%
VGC−400
acc
NZ_CP031277.1
pident
70
80
90
100
Average identity=98.28%
VGC−410
acc
NZ_CP023966.1
pident
80
90
100
Average identity=98.93%
VGC−404
acc
NZ_CP042341.1
pident
80
90
100
Average identity=98.95%
VGC−414
pident
80
90
100
acc
NZ_CP031277.1
Average identity=98.01%

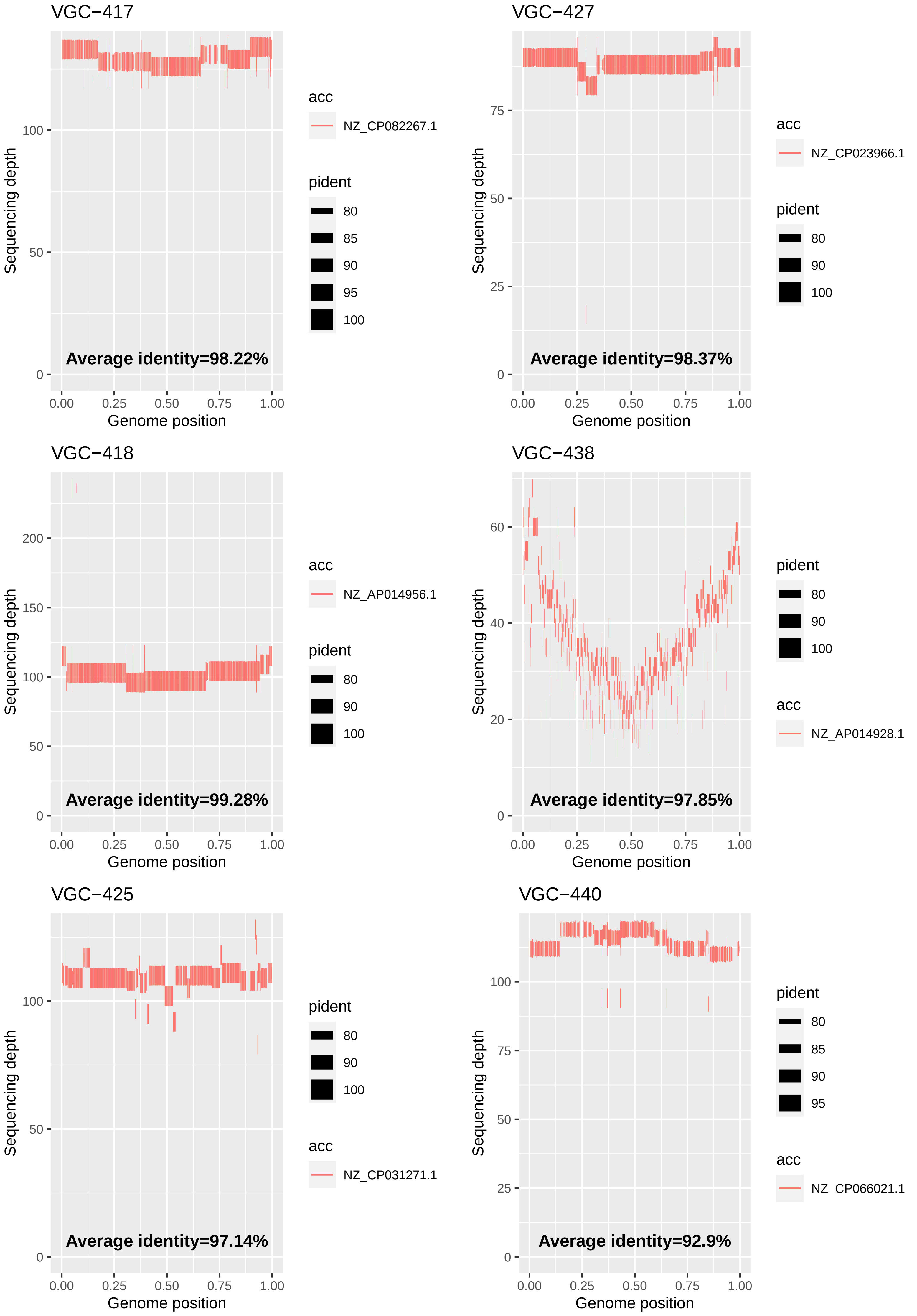
VGC−417
Sequencing depth
Genome position
Average identity=98.22%
acc
NZ_CP082267.1
pident
80
85
90
95
100
VGC−427
Average identity=98.37%
NZ_CP023966.1
VGC−418
Average identity=99.28%
NZ_AP014956.1
VGC−438
Average identity=97.85%
NZ_AP014928.1
VGC−425
Average identity=97.14%
NZ_CP031271.1
VGC−440
Average identity=92.9%
NZ_CP066021.1

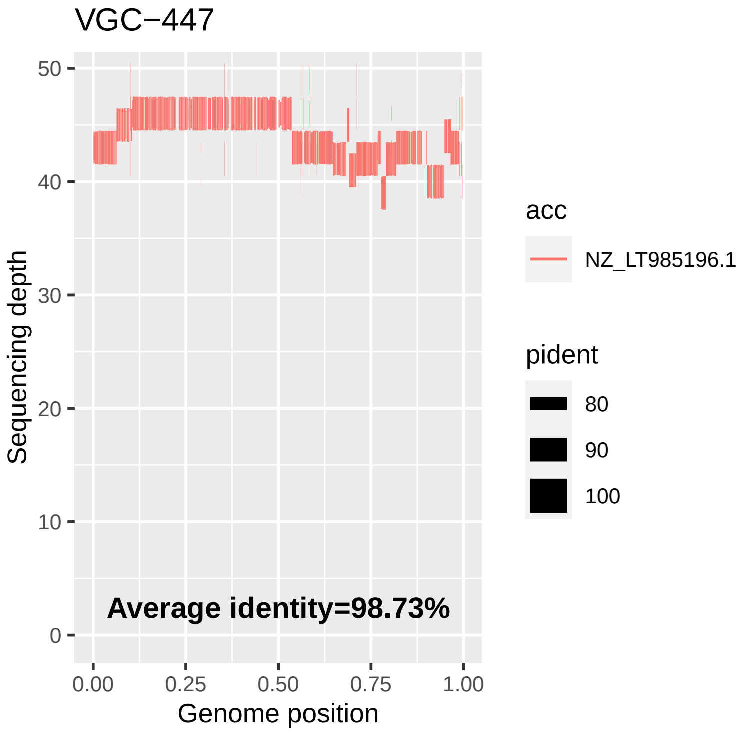

VGC−447
50
40
30
20
10
0
Sequencing depth
0.00
0.25
0.50
0.75
1.00
Genome position
Average identity=98.73%
acc
NZ_LT985196.1
pident
80
90
100

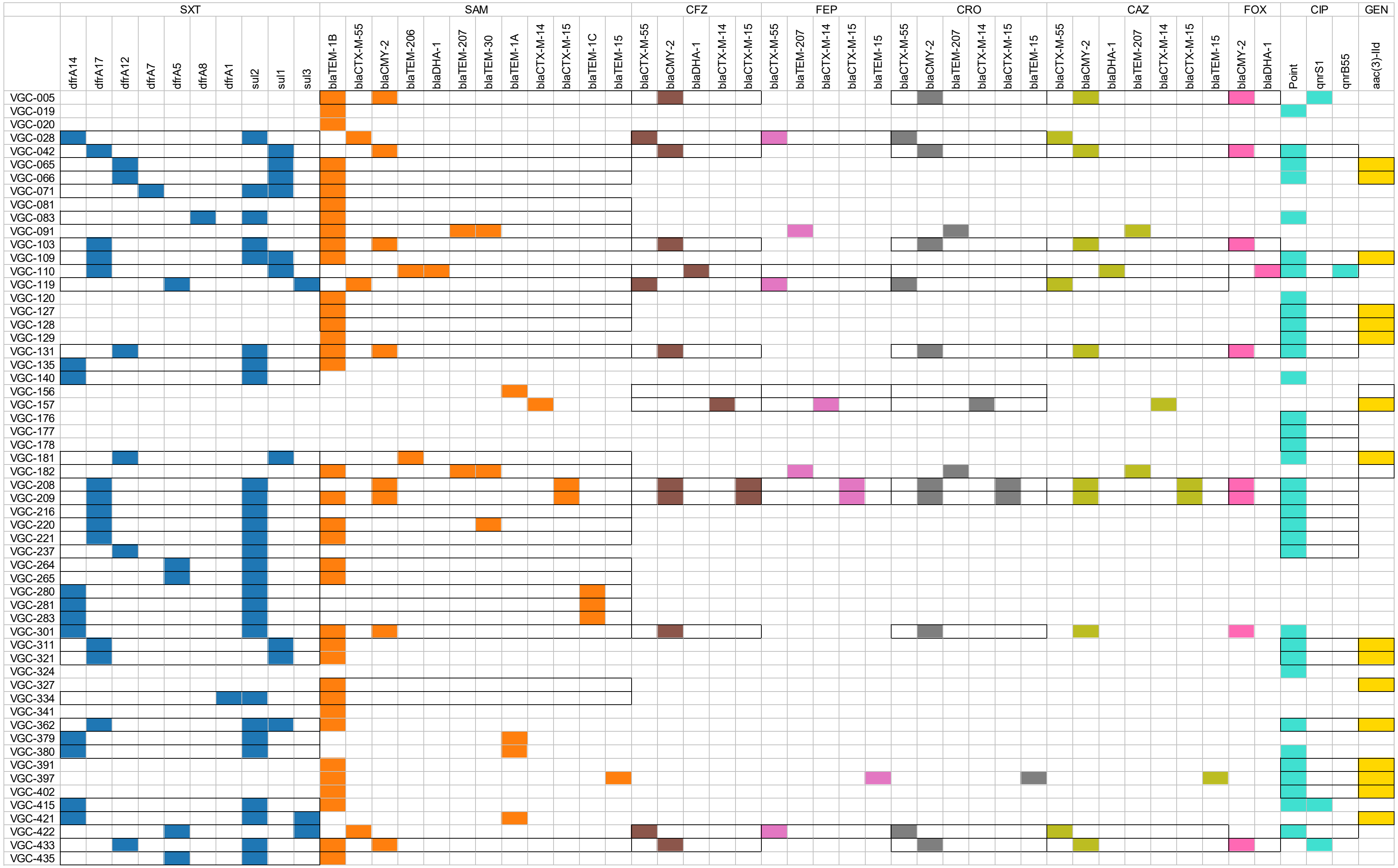
SXT
SAM
CFZ
FEP
CRO
CAZ
FOX
CIP
GEN
dfrA14
dfrA17
dfrA12
dfrA7
dfrA5
dfrA8
dfrA1
sul2
sul1
sul3
blaTEM-1B
blaCTX-M-55
blaCMY-2
blaTEM-206
blaDHA-1
blaTEM-207
blaTEM-30
blaTEM-1A
blaCTX-M-14
blaCTX-M-15
blaTEM-1C
blaTEM-15
blaCTX-M-55
blaCMY-2
blaDHA-1
blaCTX-M-14
blaCTX-M-15
blaCTX-M-55
blaTEM-207
blaCTX-M-14
blaCTX-M-15
blaTEM-15
blaCTX-M-55
blaCMY-2
blaTEM-207
blaCTX-M-14
blaCTX-M-15
blaTEM-15
blaCTX-M-55
blaCMY-2
blaDHA-1
blaTEM-207
blaCTX-M-14
blaCTX-M-15
blaTEM-15
blaCMY-2
blaDHA-1
Point
qnrS1
qnrB55
aac(3)-IId
VGC-005
VGC-019
VGC-020
VGC-028
VGC-042
VGC-065
VGC-066
VGC-071
VGC-081
VGC-083
VGC-091
VGC-103
VGC-109
VGC-110
VGC-119
VGC-120
VGC-127
VGC-128
VGC-129
VGC-131
VGC-135
VGC-140
VGC-156
VGC-157
VGC-176
VGC-177
VGC-178
VGC-181
VGC-182
VGC-208
VGC-209
VGC-216
VGC-220
VGC-221
VGC-237
VGC-264
VGC-265
VGC-280
VGC-281
VGC-283
VGC-301
VGC-311
VGC-321
VGC-324
VGC-327
VGC-334
VGC-341
VGC-362
VGC-379
VGC-380
VGC-391
VGC-397
VGC-402
VGC-415
VGC-421
VGC-422
VGC-433
VGC-435

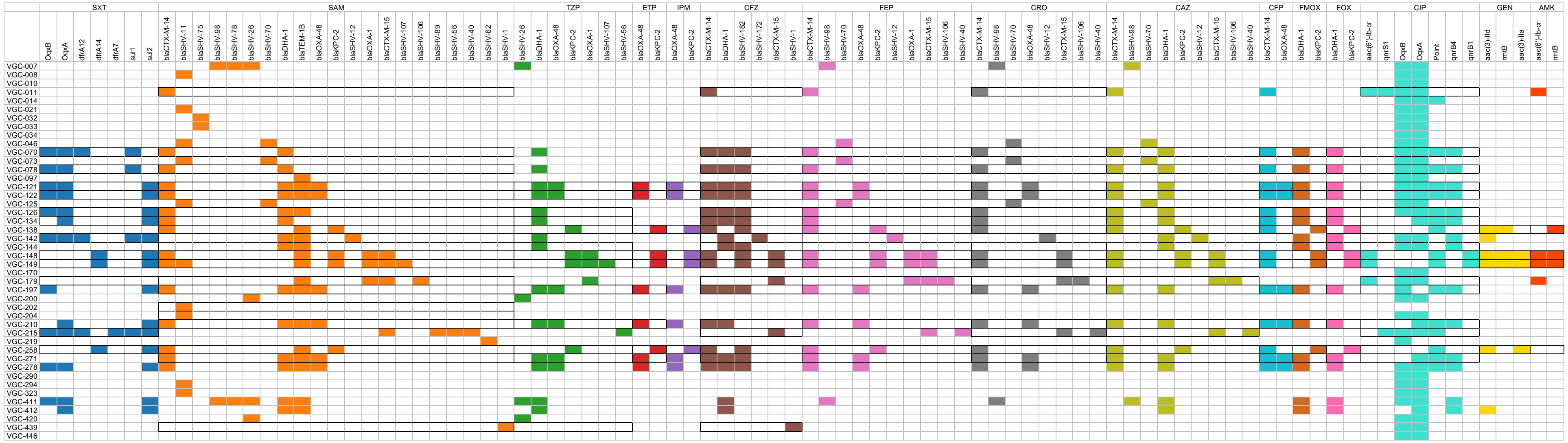
SXT
OqxB OqxA dfrA12 dfrA14 dfrA7 sul1 sul2
SAM
blaCTX-M-14 blaSHV-11 blaSHV-75 blaSHV-98 blaSHV-78 blaSHV-26 blaSHV-70 blaDHA-1 blaTEM-1B blaOXA-48 blaKPC-2 blaSHV-12 blaOXA-1 blaCTX-M-15 blaSHV-107 blaSHV-106 blaSHV-89 blaSHV-56 blaSHV-40 blaSHV-62 blaSHV-1
TZP
blaSHV-26 blaDHA-1 blaOXA-48 blaKPC-2 blaOXA-1 blaSHV-107 blaSHV-56
ETP
blaOXA-48 blaKPC-2
IPM
blaOXA-48 blaKPC-2
CFZ
blaCTX-M-14 blaDHA-1 blaSHV-182 blaSHV-172 blaCTX-M-15 blaSHV-1
FEP
blaCTX-M-14 blaSHV-98 blaSHV-70 blaOXA-48 blaKPC-2 blaSHV-12 blaOXA-1 blaCTX-M-15 blaSHV-106 blaSHV-40
CRO
blaCTX-M-14 blaSHV-98 blaSHV-70 blaOXA-48 blaSHV-12 blaCTX-M-15 blaSHV-106 blaSHV-40
CAZ
blaCTX-M-14 blaSHV-98 blaSHV-70 blaDHA-1 blaKPC-2 blaSHV-12 blaCTX-M-15 blaSHV-106 blaSHV-40
CFP
blaCTX-M-14 blaOXA-48
FMOX
blaDHA-1 blaKPC-2
FOX
blaDHA-1 blaKPC-2
CIP
aac(6')-Ib-cr qnrS1 OqxB OqxA Point qnrB4 qnrB1
GEN
aac(3)-IId rmtB aac(3)-IIa
AMK
aac(6')-Ib-cr rmtB
VGC-007
VGC-008
VGC-010
VGC-011
VGC-014
VGC-021
VGC-032
VGC-033
VGC-034
VGC-046
VGC-070
VGC-073
VGC-078
VGC-097
VGC-121
VGC-122
VGC-125
VGC-126
VGC-134
VGC-138
VGC-142
VGC-144
VGC-148
VGC-149
VGC-170
VGC-179
VGC-197
VGC-200
VGC-202
VGC-204
VGC-210
VGC-215
VGC-219
VGC-258
VGC-271
VGC-278
VGC-290
VGC-294
VGC-323
VGC-411
VGC-412
VGC-420
VGC-439
VGC-446

	PEN		OX	CLI				ERY				
	blaZ	mecA	mecA	lnu(A)	erm(A)	erm(C)	erm(B)	msr(A)	erm(A)	mph(C)	erm(C)	erm(B)
VGC-022												
VGC-037												
VGC-043												
VGC-051												
VGC-052												
VGC-055												
VGC-093												
VGC-094												
VGC-190												
VGC-203												
VGC-233												
VGC-239												
VGC-242												
VGC-251												
VGC-252												
VGC-269												
VGC-270												
VGC-308												
VGC-309												
VGC-336												
VGC-339												
VGC-340												
VGC-343												
VGC-344												
VGC-353												
VGC-356												
VGC-358												
VGC-359												
VGC-372												
VGC-389												
VGC-403												
VGC-406												
VGC-407												
VGC-409												
VGC-413												

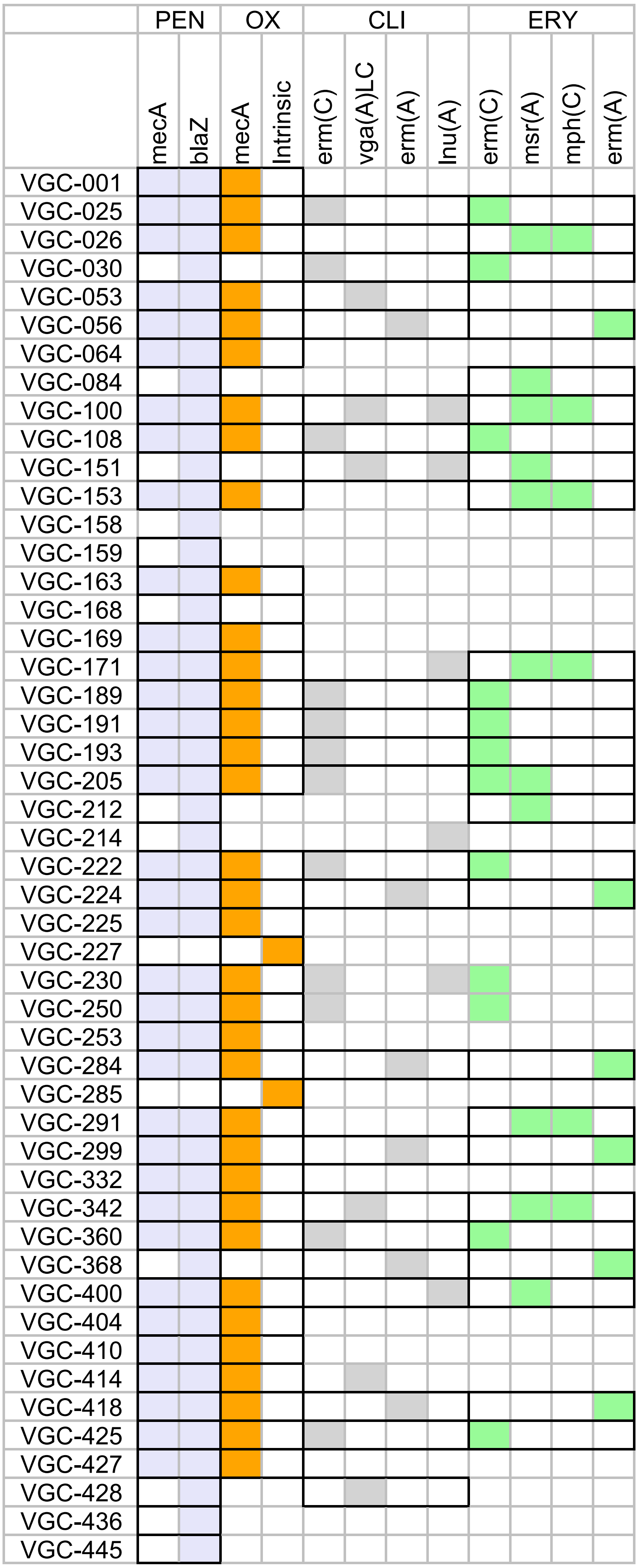
PEN
OX
CLI
ERY
mecA
blaZ
mecA
Intrinsic
erm(C)
vga(A)LC
erm(A)
lnu(A)
erm(C)
msr(A)
mph(C)
erm(A)
VGC-001
VGC-025
VGC-026
VGC-030
VGC-053
VGC-056
VGC-064
VGC-084
VGC-100
VGC-108
VGC-151
VGC-153
VGC-158
VGC-159
VGC-163
VGC-168
VGC-169
VGC-171
VGC-189
VGC-191
VGC-193
VGC-205
VGC-212
VGC-214
VGC-222
VGC-224
VGC-225
VGC-227
VGC-230
VGC-250
VGC-253
VGC-284
VGC-285
VGC-291
VGC-299
VGC-332
VGC-342
VGC-360
VGC-368
VGC-400
VGC-404
VGC-410
VGC-414
VGC-418
VGC-425
VGC-427
VGC-428
VGC-436
VGC-445
